# Chromosomal Position of Ribosomal Protein Genes Affects Long-Term Evolution of Vibrio cholerae

**DOI:** 10.1128/mbio.03432-22

**Published:** 2023-03-02

**Authors:** Leticia Larotonda, Damien Mornico, Varun Khanna, Joaquín Bernal-Bayard, Jean-Marc Ghigo, Marie-Eve Val, Diego Comerci, Didier Mazel, Alfonso Soler-Bistué

**Affiliations:** a Instituto de Investigaciones Biotecnológicas “Rodolfo A. Ugalde,” IIB-IIBIO, Universidad Nacional de San Martín-CONICET, San Martín, Buenos Aires, Argentina; b Institut Pasteur, Université de Paris Cité, CNRS UMR 3525, Unité Plasticité du Génome Bactérien, Paris, France; c Institut Pasteur, Université de Paris Cité, Bioinformatics and Biostatistics Hub, Paris, France; d Institut Pasteur, Université de Paris Cité, CNRS UMR 6047, Genetics of Biofilms Laboratory, Paris, France; e Departamento de Genética, Facultad de Biología, Universidad de Sevilla, Sevilla, Spain; Vanderbilt University

**Keywords:** experimental evolution, genomics, growth rate, ribosomal protein, Vibrio cholerae

## Abstract

It is unclear how gene order within the chromosome influences genome evolution. Bacteria cluster transcription and translation genes close to the replication origin (*oriC*). In Vibrio cholerae, relocation of *s10-spc-α* locus (S10), the major locus of ribosomal protein genes, to ectopic genomic positions shows that its relative distance to the *oriC* correlates to a reduction in growth rate, fitness, and infectivity. To test the long-term impact of this trait, we evolved 12 populations of V. cholerae strains bearing S10 at an *oriC*-proximal or an *oriC*-distal location for 1,000 generations. During the first 250 generations, positive selection was the main force driving mutation. After 1,000 generations, we observed more nonadaptative mutations and hypermutator genotypes. Populations fixed inactivating mutations at many genes linked to virulence: flagellum, chemotaxis, biofilm, and quorum sensing. Throughout the experiment, all populations increased their growth rates. However, those bearing S10 close to *oriC* remained the fittest, indicating that suppressor mutations cannot compensate for the genomic position of the main ribosomal protein locus. Selection and sequencing of the fastest-growing clones allowed us to characterize mutations inactivating, among other sites, flagellum master regulators. Reintroduction of these mutations into the wild-type context led to a ≈10% growth improvement. In conclusion, the genomic location of ribosomal protein genes conditions the evolutionary trajectory of V. cholerae. While genomic content is highly plastic in prokaryotes, gene order is an underestimated factor that conditions cellular physiology and evolution. A lack of suppression enables artificial gene relocation as a tool for genetic circuit reprogramming.

## INTRODUCTION

In all known life forms, DNA stores genetic information in a long polymer whose length exceeds cell dimensions by thousands of times (1). As a consequence, the genetic material is highly packed by specific factors that confer its structure and position within the cell (2–5). In bacteria, there is relatively little knowledge on the physiological role of spatial organization of the chromosome. The simplest model to tackle this issue is the bacterial cell due to its relatively small genome size and chromosome number. The bacterial chromosome is tightly organized within the confined space of the cytoplasm and throughout the cell cycle (6–13). This is physiologically relevant, since DNA simultaneously serves as a template for replication, transcription, segregation, and repair of the genetic material (1, 4, 14). Bacterial chromosomes have a single replication origin (*oriC*) from which DNA duplication begins bidirectionally until the terminal region (*ter*) where both replication forks meet. This configuration organizes the genome into two generally equally sized replichores along the *ori-ter* axis (6, 15, 16). Recent studies suggest that gene order contributes to link genome structure to cell physiology (14, 16–20). The genomic position of key gene groups is widely conserved along the *ori-ter* axis following the temporal pattern of gene expression (17, 18, 21, 22). Thus, coding sequences expressed during the exponential phase are physically associated with the *oriC* region, while the genes transcribed in stress situations or in stationary-phase cluster close to the *ter* region (17, 21, 23–26). Consistently, it was recently shown that some genes need a specific genomic location to achieve their function (16). In most cases, the genomic position of a gene conditions its genome-wide copy number, since those located close to *oriC* are overrepresented compared to those close to the *ter* region during DNA replication (16, 27). This differential template abundance, called the gene dosage effect, leads to increased expression of genes located in the proximity of *oriC*, although this might be more complex (28, 29). The *ori-ter* template abundance gradient is particularly important in fast-growing bacteria during steady-state growth when cells display generation times shorter than the time required for complete genome replication. Under these conditions, successive replication rounds overlap, a mechanism called multifork replication (MFR) (16, 27). In fact, many essential and highly expressed genes are clustered in this chromosomal region (6, 24, 30–32). In parallel, some other genes require a precise location to function, independently of their dosage (33, 34). Other coding sequences, particularly long highly expressed operons affect the chromosomal spatial structure (10, 11), suggesting an interplay between gene order and chromosome structure (35).

Vibrio cholerae is a human pathogen of prime importance able to colonize the intestine causing cholera disease, which kills around 100,000 persons yearly (36). This microorganism is a common inhabitant of estuarine environments often associated with plankton (37, 38). It is a highly motile bacterium thanks to a single, sheathed polar flagellum. The biogenesis of the flagellum depends on a regulation cascade whose master regulator is the FlrA protein (39). V. cholerae also alternates from a mobile to a sessile lifestyle by forming structured biofilms, a key trait for both parts of V. cholerae’s life cycle (40–42). Importantly, flagellum-mediated swimming, chemotaxis, biofilm formation, colonization, and virulence are intimately related processes connected through complex regulatory cascades (40, 43). In particular, flagellum-mediated motility and biofilm formation are inversely regulated by the concentration of the secondary messenger molecule cyclic di-GMP (c-di-GMP) (43–45). This molecule is in turn modulated by proteins carrying GGDEF and/or EAL domains displaying diguanylate cyclase and phosphodiesterase activity, respectively (43–46).

V. cholerae displays a short doubling time (~16 min) and high replication-associated gene dosage effects (24). It is also a model of bacteria bearing multiple chromosomes, since it harbors a main chromosome (Chr1) of 2.96 Mbp and a 1.07-Mbp secondary replicon (Chr2) (47). Replication of both chromosomes is coordinated. Chr1 first starts its replication, while Chr2 fires its replication origin (*ori2*) only after two-thirds of Chr1 is duplicated, and both chromosomes complete DNA synthesis synchronously (48). Nevertheless, replication-dependent gene-dosage effects exist in both replicons (30, 49, 50), while their relative timing modulates the gene dosage difference between both replicons. In *Vibrionaceae*, transcription and translation genes map close to the *oriC* of Chr1 (*ori1*) (31, 49).

We have previously studied the chromosomal position of ribosomal protein (RP) genes to uncover the link between gene order and cell physiology (24, 51). For this, we manipulated the genomic location of V. cholerae major ribosomal protein locus *s10-spc-α* (S10), a 13-kbp array of essential genes highly conserved among prokaryotes and also in eukaryotic organelles (52, 53). By relocating the S10 locus throughout both chromosomes (49), we generated a S10 *movant* strain set (i.e., isogenic strains in which the genomic position of the locus of interest is altered) whose growth rate, infectivity, and fitness decreased as a function of the distance between S10 and *ori1*. Relocation to the Chr2 showed a detrimental effect accordingly. These phenotypes were caused by the reduction of S10 genome-wide copy number that were mostly consequence of macromolecular crowding alterations rather than a deficit in the capacity to synthesize proteins (49, 50, 54). Further increasing S10 copy number was either deleterious or neutral. Overexpression of nonribosomal protein genes within S10 could not rescue the growth rate, indicating that these alterations were exclusively due to ribosomal protein genes (54). Genomic position of S10 maximizes cell physiology and fitness throughout both parts of V. cholerae life cycle. Meanwhile, S10 is close to *ori1* among all *Vibronaceae*. Since this clade diverged from enterobacteria some 500 million years ago (55), such genome positioning is likely to be the result of selection in the long run rather than a trait acquired through genetic drift. In the last years, long-term evolution experiments emerged as the approach of choice to directly assess evolutionary trajectories of (micro)organisms (56–59).

In this work, we showed that the genomic position of the major ribosomal protein locus conditions V. cholerae evolution in the long term. Toward this aim, we experimentally evolved strains in which the S10 locus was either placed at its original position or at the terminal region of the chromosome. We showed that the growth rate reduction observed in the latter case persisted over 1,000 generations of evolution, providing experimental support to the biased genomic position of these genes. In particular, we observed that, among other mutations, increased biofilm formation and growth rate occur at the expense of motility during *in vitro* environment evolution. However, no mutation could suppress S10 genome position, a finding that provides key evidence showing the importance of the long-term impact of gene order on genome evolution. We also provide novel evidence and observations on how V. cholerae evolves *in vitro*.

## RESULTS

### V. cholerae evolution experiments.

**FIG 1:**
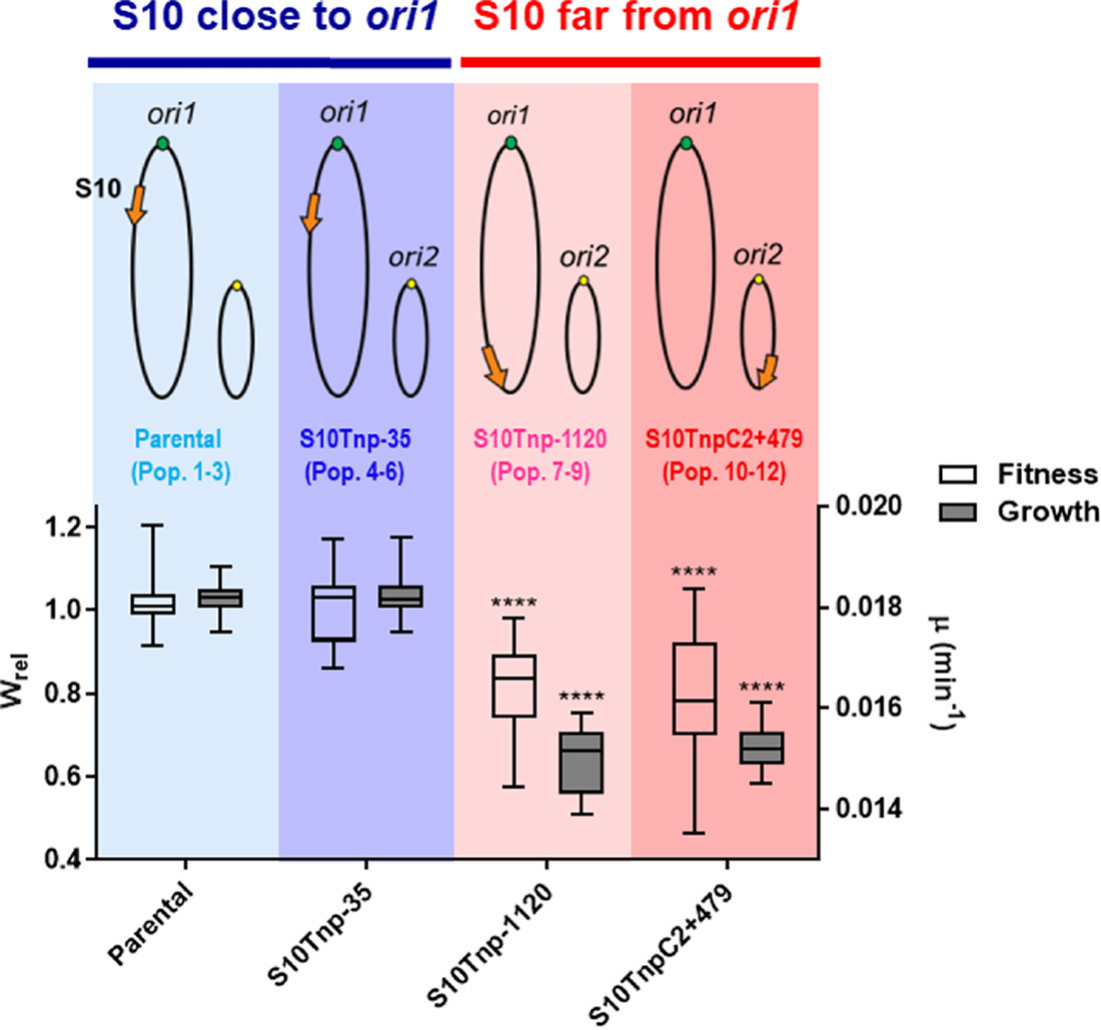
Genotype and associated phenotype of the evolved strains at the start of the experiment. Genome structure of the parental, the movant strains subjected to long-term evolution for 1,000 generations. Chromosome I and II are depicted as ovals. The orange arrow represents S10 at its genomic position with respect to *ori1* (green dot) or *ori2* (yellow dot). Colors are strain specific, and we indicate the population numbering assigned. Strains in which S10 is close to *ori1* are colored with shades of blue, while those having the locus in a distal position are colored with shades of red. The maximum growth rate (μ, gray boxes, right axis) and the relative fitness (*W*_rel_, white boxes, left axis) with respect to the parental strain obtained in previous works were plotted for each strain. The μ obtained from the slope of the growth curve during exponential phase in fast-growing conditions (118). The fitness of each strain was measured by pairwise competition against a parental strain. The results are shown in standard box-and-whisker plots showing the medians, minima, and maxima. The detailed calculations of these parameters can be found in references 49 and 50. Statistical significance was analyzed using nonparametric tests and the Dunn test for multiple comparisons of the means obtained for each strain. ****, *P* < 0.0001.

Since we aimed at understanding how the genomic location of S10 could condition long-term evolution, we conducted the experimental evolution of six populations in which the locus is close to *ori1* (blue) and six populations in which S10 resides at termini of the chromosomes (red) for 1,000 generations (Fig. 1, upper panel). For the six populations harboring S10 close to *ori1*, three populations were derived from a strain in which S10 was not transposed (49) (Fig. 1; Table S1, populations P1 to P3). The other three populations were evolved from S10Tnp-35, a movant in which the S10 locus was slightly relocated (Fig. 1; Table S1, populations P3 to P6) that displays no fitness or growth rate defect (Fig. 1). It is isogenic with respect to the rest of the movants and shows only eight genes that are differentially expressed with respect to the parental strain (54). It is a control that any possible genetic change associated with S10 relocation *per se* could affect the evolution of V. cholerae.

10.1128/mbio.03432-22.6 TABLE S1 Full list of plasmids and bacteria strains used in this study. Download Table S1, PDF file, 0.1 MB. Copyright © 2023 Larotonda et al. 2023 Larotonda et al. https://creativecommons.org/licenses/by/4.0/ This content is distributed under the terms of the Creative Commons Attribution 4.0 International license.

In parallel, we evolved six populations where S10 was relocated far from *ori1*. We used three isolated clones of S10Tnp-1120 (Populations P7 to P9) and three clones of S10TnpC2 + 479 (Table S1, populations P10 to P12) in which S10 was moved to the *ter* region of Chr1 and Chr2, respectively (Fig. 1, upper panel, red). These are the most phenotypically affected movants, displaying an ~15% loss of maximal growth rate and lower fitness than the parental strain (Fig. 1, lower panel, red) (50).

Due to the fast growth of V. cholerae, we passaged them twice a day in lysogeny broth (LB) supplemented with streptomycin (Str) to avoid contamination. The populations were regularly frozen. We also isolated single clones at 250 and 1,000 generations for later analysis.

### Evolved V. cholerae populations increase their biofilm formation capacity.

**FIG 2:**
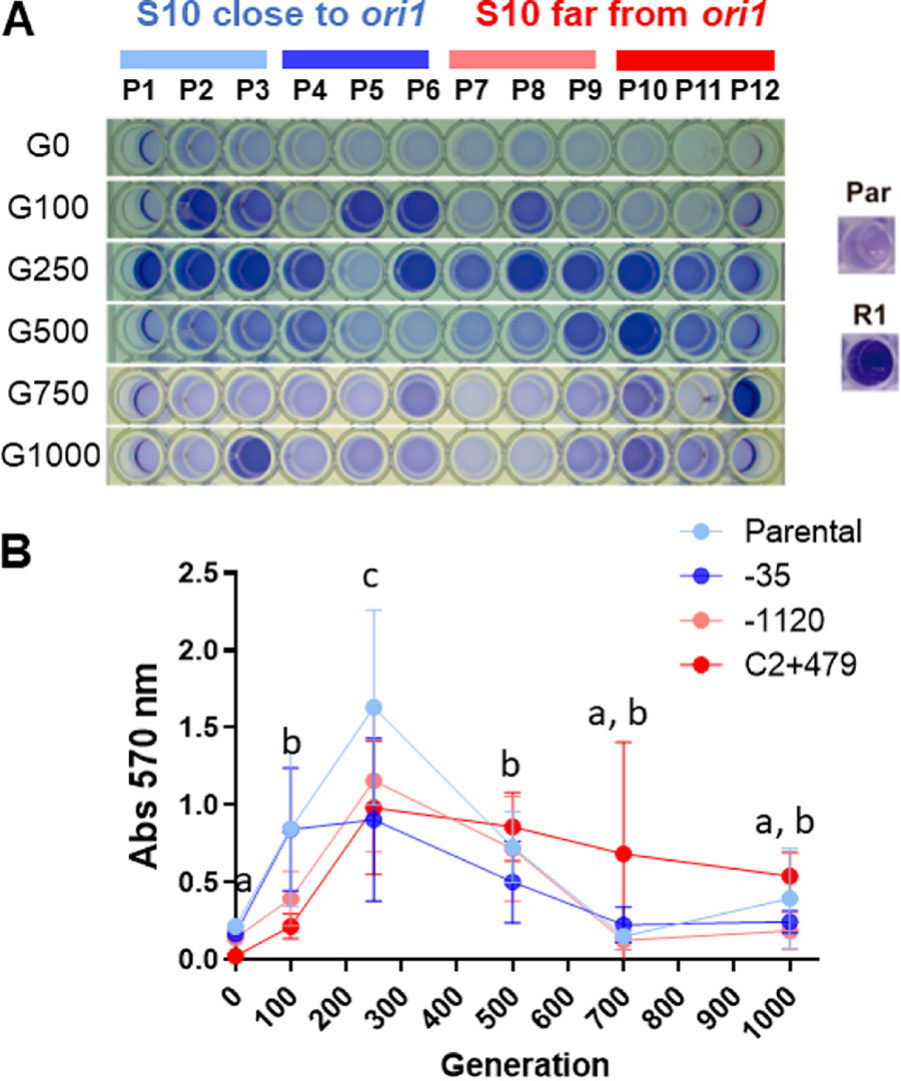
Evolution of biofilm formation capacity throughout the evolution experiment. Biofilm formation phenotype of 12 evolved populations from strains in which S10 is close to ori1 (shades of blue) or at a distal position from replication origin (shades of red) is shown. (A) Representative wells in which cells were stained with crystal violet are shown for P1 to P12 at different time points. Par, parental strain; R1, rugose clone used as negative and positive controls for biofilm-forming capacity. (B) The crystal violet stain was quantified for all populations throughout generations. The parental (light blue, P1 to P3), S10Tnp-35 (dark blue, P4 to P6), S10Tnp-1120 (light red, P7 to P9), and S10TnpC2 + 479 (red, P10 to P12) movants populations values were averaged for simplicity (Fig. S2 shows the disaggregated data). The data are averages of the means from three independent experiments done in quadruplicate. Statistical significance was analyzed by ordinary two-way analysis of variance (ANOVA) two-tailed test with main effects only. Then, Tukey’s test was done to compare the mean Abs_570_ values. The factors analyzed were strains and generations. There were no significant differences in Abs_570_ values between strains. Letters denote statistically significant differences in the means between generations (*P* < 0.05).

First, we noticed the emergence of strong biofilms in the Erlenmeyer flasks as early as generation 81 (G81). By generation G100, most populations displayed strong biofilms at the air-liquid interface and/or dense cell aggregates in the medium (Fig. S1A). By G250, biofilms were present in all populations. Accompanying this phenomenon, colonies of abnormal morphology appeared, particularly rugose colonies (Fig. S1B). The subculture of isolated smooth (S) and rugose (R) colonies showed that only the latter formed strong biofilms in the flasks (Fig. S1C). To quantitatively address the biofilm-forming capacity of the populations throughout experimental evolution, we cultured them statically in 96-well polystyrene plates, washed them, and then stained the wells with crystal violet at G0, G100, G250, G500, G700, and G1000 (Fig. 2). As positive and negative controls, we analyzed R1, a rugose clone isolated from P1 at G1000 (see above), and the ancestral parental strain, respectively. At the beginning of the experiment (G0), the levels of crystal violet stain were minimal. Then, biofilm-formation capacity increased rapidly in all populations. On average, the biofilm formation capacity reached the maximum at ~G250 (Fig. 2), although some specific populations displayed an earlier peak near ~G100 (Fig. S2). After G250, biofilm formation capacity decreased in all evolved populations without going back to initial G0 baseline levels. The emergence of rugose colonies paralleled the evolution of biofilm-forming capacity (Fig. S1D). All populations displayed a similar trend, suggesting that it was selected throughout the experiment independently of S10 genomic location (Fig. 2; Fig. S2), being a common trait for V. cholerae *in vitro* evolution.

10.1128/mbio.03432-22.1 FIG S1 Qualitative characterization of biofilm formation during the laboratory evolution experiment. (A) General aspects of a population displaying strong biofilms (P3) and a population lacking this trait (P1) forming after 114 generations. (B) Macroscopic aspect of a smooth and a rugose colony in LB agar. (C) Erlenmeyer cultures of the parental strain at G0 and rugose and smooth clones from P1 and P7 isolated at G1000. R strains showed strong biofilms in the liquid air-interphase absent in the other clones. (D) The proportion of rugose clones through the generations is shown for four representative populations. The inset shows a representative photograph of P1 straked on LB agar with at maximum (upper panel, G250) and a minimum (lower panel, G100) proportion of R clones. Download FIG S1, PDF file, 0.1 MB. Copyright © 2023 Larotonda et al. 2023 Larotonda et al. https://creativecommons.org/licenses/by/4.0/ This content is distributed under the terms of the Creative Commons Attribution 4.0 International license.

10.1128/mbio.03432-22.2 FIG S2 Quantitative characterization of biofilm formation throughout the LTEE. Crystal violet staining assays for all populations throughout the LTEE. The parental (shades of violet, P1 to P3) and S10Tnp-35 (shades of cyan, P4 to P6), S10Tnp-1120 (shades of red, P7 to P9), and S10TnpC2 + 479 (shades of blue, P10 to P12). The data are the averages of the means from three independent experiments. Download FIG S2, PDF file, 0.01 MB. Copyright © 2023 Larotonda et al. 2023 Larotonda et al. https://creativecommons.org/licenses/by/4.0/ This content is distributed under the terms of the Creative Commons Attribution 4.0 International license.

### Evolved populations display a growth rate increase.

**FIG 3:**
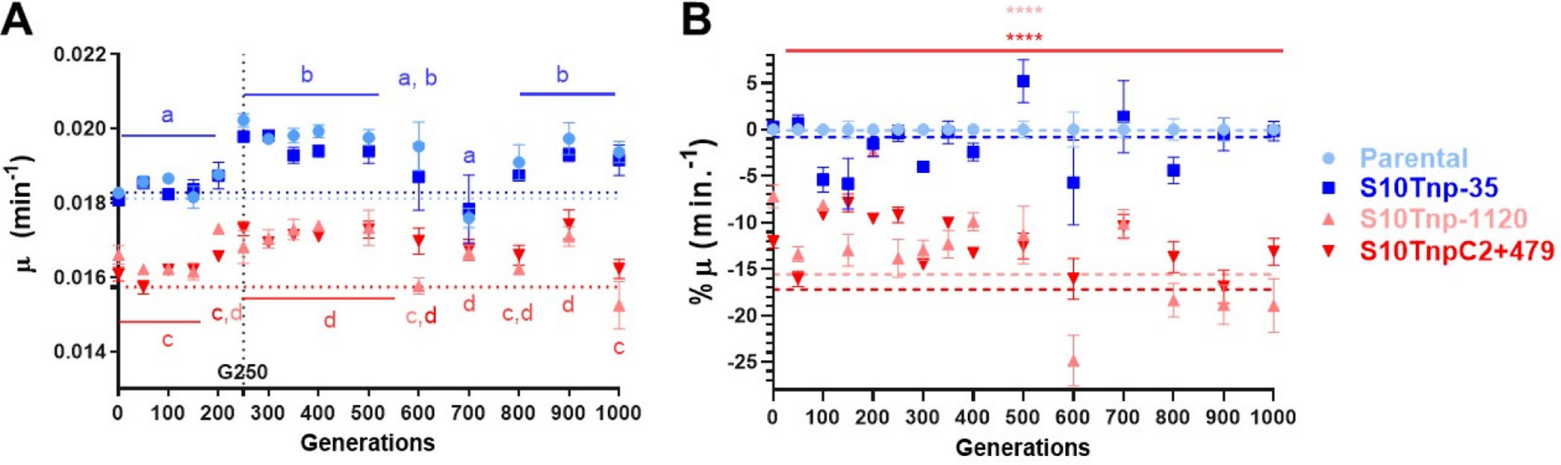
S10 genomic location affects growth rate evolution in the long-term. (A) V. cholerae increases its growth rate throughout experimental evolution. At every time point, the populations from the frozen “fossil record” were subjected to automated growth curves and relativized to their ancestral strains. The strains in which S10 is close to *ori1* are depicted in shades of blue, while those were bearing the locus at an *ori1* distal location are shown in shades of red. We show the averages of parentals (light blue), S10Tnp-35 (blue), S10Tnp-1120 (light red), and S10TnpC2 + 479 (red) strains. The graph shows the average of at least two experiments done by triplicate of μ ± standard error of the mean (SEM) as a function of the generations elapsed. The average μ from the ancestral strains is shown as dotted lines for reference. Individual populations are plotted in Fig. S3A. Statistical significance was assessed using ordinary two-way ANOVA two-tailed test with main effects only. Then, Tukey’s test was done to compare the mean of μ. Letters denote groups being statistically different. (B) Growth rate defect of movants persists through 1,000 generations of evolution. The obtained μ for each population (P4 to P12) was relativized to growth rate of the Parental populations (P1 to P3) for each time point. Then, the mean with SEM of percentage of μ variation was plotted as a function of generations. The dotted lines represent the average deviation from growth of the parental for each ancestral strain already shown in Fig. 1 (0% for parental, 0.79% for S10Tnp-35 and −15.5% for S10Tnp-1120 and −17.2% for S10TnpC2 + 479). Statistical significance against parental strain was assessed using ordinary two-way ANOVA two-tailed test with main effects only. Then, Tukey’s test was done to compare the means. Across all generations, the percentage of μ is significantly lower in strains in which S10 locus is far from *ori1*, and S10Tnp-35 is not significantly different than the parental strain. ****, *P* < 0.0001.

Previous studies showed that bacterial populations increased their fitness over time compared to the ancestral clone (58, 60). We employed maximum growth rate (μ) for fitness estimation, since in previous works, fitness measured by pairwise competitions and μ changed similarly as a function of S10 genomic location (50). The evolved populations were subjected to automated growth curves. For this, at each time point, the 12 populations were diluted 1 in 1,000 and cultured in LB at 37°C measuring optical density at 620 nm (OD_620_) in triplicate at least twice. In each experiment, the four ancestral strains (parental, S10Tnp + 35, S10Tnp-1120, and S10TnpC2 + 479; Table S1) were used as a control. The value of μ was calculated from the growth curves and relativized to the parental strains to remove variations between experiments. Then, we plotted the maximum growth rate as a function of elapsed generations (Fig. 3A; Fig. S3A). During the first 150 generations, μ remained stable among populations. As expected (49, 50), populations derived from strains in which S10 is close to *ori1* (P1 to P6, blue) grew ~15% faster than those originated from the most affected movants (P7 to P12, red). After this period, between G150 and G250, populations show a sudden μ increment. All populations increased their growth rate throughout experimental evolution, but this rise occurred in a stepwise manner, with the most notable occurring at G250. After G500, the alterations in growth rate become noisier, since populations originating from the same strain tend to diverge (Fig. S3A and C). Despite the general μ increment through generations, the differences between populations evolving from faster strains in which S10 is close to *ori1* (P1 to P6) and slower ones in which S10 was relocated to chromosome termini (P7 to P12) persisted throughout the experiment (Fig. 3B; Fig. S3A to C). The evolutionary trajectory of this trait was similar for all of them (Fig. S3D). Importantly, we did not notice suppressor mutants compensating growth rate deficit in P7 to P12. Hence, mutations able to compensate genomic position of S10 locus are very difficult to achieve, at least in this time frame. This strongly suggests that the S10 genomic location conditions growth rate throughout experimental evolution, strongly indicating that the position of the main ribosomal protein locus determines fitness in the long run.

10.1128/mbio.03432-22.3 FIG S3 Growth rate evolution through the generations elapsed. (A) This figure is similar to Fig. 3A but shows the results for each population without averaging them. At every time point, the populations from the frozen fossil record were subjected to automated growth curves and relativized to their ancestral strains. The populations where S10 is close to *ori1* (P1 to P6) are depicted in shades of blue, while those were bearing the locus at an *ori1* distal location are shown in shades of red (P7 to P12). The colors used are as follows: light red for parental populations (P1 to P3), dark red for S10Tnp-35 populations (P4 to P6), light blue for S10Tnp-1120 (P7 to P9), and dark blue for S10TnpC2 + 479 (P10 to 12, blue) strains. These colors are maintained throughout the figure. The graph shows the averages of at least two experiments done by triplicate of μ ± standard deviation (SD) as a function of the generations elapsed. The average μ from the ancestral strains is shown as dotted lines for reference. (B) This figure is similar to Fig. 3B but shows the results for each population without averaging them. The obtained μ for each population was relativized to average growth rate of the parental populations (P1 to P3) for each time point. Then, the mean ± SD of percentage of μ variation was plotted as a function of generations. The dotted lines represent the average deviation from growth of the parental for each ancestral strain already shown in Fig. 1 (0% for parental, 0.79% for S10Tnp-35, −15.5% for S10Tnp-1120, and 17.2% for S10TnpC2 + 479). (C) The obtained μ for each population was divided by the growth rate of its own ancestral strain. Then, relative growth was plotted as a function of generations. The dotted line indicates 1, which represents no change with respect to the ancestral strain founding the population. (D) The raw data of μ as a function of generations elapsed are shown with no relativization. The μ of each ancestral founding clone is shown at each time point as black squares for the parental, light grey circles for S10Tnp-35, dark grey for S10Tnp-1120, and white circles for S10TnpC2 + 479. These data were used to filter noise due to growth variations between time points (see Materials and Methods). The trend is similar to Fig. 3A and Fig. S3A, showing that relativizing growth parameters to the parental strain did not alter the results of the experiment. Download FIG S3, PDF file, 0.10 MB. Copyright © 2023 Larotonda et al. 2023 Larotonda et al. https://creativecommons.org/licenses/by/4.0/ This content is distributed under the terms of the Creative Commons Attribution 4.0 International license.

### Positive selection drives genome dynamics in the first 250 generations of experimental evolution.

To better characterize mutations occurring through the experiment, we deep-sequenced each ancestral strain as a reference and then each population at G250 and G1000. We speculated that mutations at G250 would correlate to the earlier increase in growth rate and the emergence of biofilm-formation capacity.

**FIG 4:**
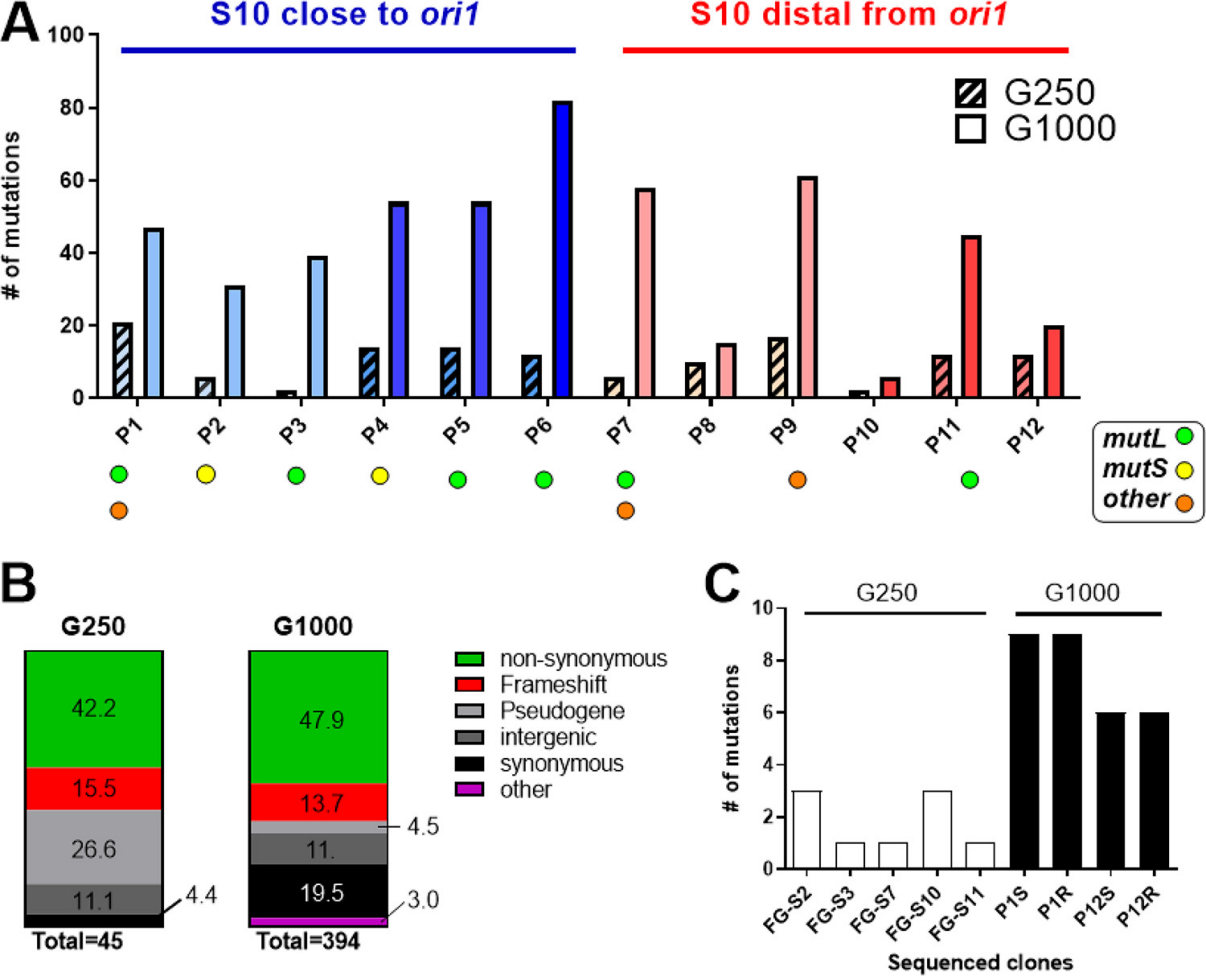
Evolutionary trajectories of the evolved populations. (A) The graph shows the number mutations fixed (present in at least of 25% of the population). For each population, the patterned and plain bars indicate the mutations detected at G250 and G1000, respectively. The colors indicate the ancestral strain for the population: shades of blue for those bearing S10 close and shades of red for S10 at a distal location from *ori1* as in previous figures. The filled circles indicate populations showing mutations at the DNA repair genes: *mutL* (green), *mutS* (yellow), and others (e.g., *recBCD*, *recJ*; orange). (B) Each column represent the total of the observed mutations at G250 and G1000. The colors represent the proportion of each type of mutation observed. The numbers within the colored areas indicate the corresponding percentage. (C) Total mutations fixed in the single clones sequenced. The white columns represent the fast-growing clones (FG) isolated at G250. The dark columns represent the S and R clones isolated at G1000.

Deep sequencing of the 12 populations at G250 revealed 128 mutations observed in more than 25% of the reads obtained from each culture (Data Set S1A). Among them, we found 45 different mutations among all populations. The number of fixed DNA changes varied from 2 to 21 between each individual population (Fig. 4A). On average, each fixed mutation occurred in two or three populations, strongly indicating that they were under positive selection. Most of them (26 of 45) corresponded to nonsynonymous mutations or frameshifts of coding sequences (Fig. 4B). These changes are likely to alter or interrupt their encoded functions. Meanwhile, 17 changes (42%) occurred *a priori* in noncoding regions such as intergenic spaces and pseudogenes (Fig. 4B). We detected two (4.4%) synonymous mutations. When the mutations were grouped by coding sequences (CDS), we found that at least 17 genes suffered alterations (Data Set S1). Among them, six genes presented more than one mutation across multiple populations: *aroG*. *ompT*, *flrA*, *flrB*, *flrC*, and *tagE2* (Table 1). First, *ompT* (61) is a highly expressed gene encoding one of the main porins of V. cholerae. *tagE2* (VCA1043) was found to be altered in most of the populations. There is little evidence of its encoded function, although a recent study shows that it is an endopeptidase necessary for cell growth (62). The *aroG* gene is required for the synthesis of aromatic amino acids, although little work has been done in V. cholerae. Finally, we found single nucleotide polymorphisms (SNPs) at *flr* genes, the main regulators of flagellum biosynthesis (39, 63, 64). FlrA is the master regulator of flagellum biosynthesis that controls the expression of *flrB* and *flrC*, which in turn are downstream of the flagellar transcription hierarchy (39). We reasoned that this multiplicity of independent mutational events within the same regulation pathway suggests a fitness benefit from motility inactivation. Meanwhile, the gene order seems unaltered among all populations (Fig. S4 and S5).

**TABLE 1:** Recurrent mutations occurring on evolved V. cholerae populations after 250 generations^*a*^

Gene	Function	Population	Type of mutation^*b*^
*ompT* (VC1854)	Main porin	9, 10, 11, and 12	FS, S
*flrA* (VC2137)	Flagellar master regulator	1, 6	FS, NS
*flrB* (VC2136)	Flagellar regulator (class II)	3, 4	FS, NS
*flrC* (VC2135)	Flagellar regulator (class II)	2, 9, 10	FS, NS
*aroG* (VCA1036)	Aromatic amino acid synthesis	1, 6, 8, 9, 12	NS
*tagE2* (VCA1043)	Endopeptidase	1, 4, 5, 7, 9, 11, 12	NS

a We included genes mutated more than once in multiple populations. Data Set S1A includes the full list and details of all mutations.

b FS, frame-shift mutation; NS, nonsynonymous mutation; S, synonymous mutation.

10.1128/mbio.03432-22.4 FIG S4 Genome large rearrangement analysis of populations. The next-generation sequencing (NGS) data from the founding clone (upper panel) or populations evolved after 250 (G250) or 1,000 generations of experimental evolution (G1000) was recovered and analyzed as described under Materials and Methods. Visualization was done using the Artemis comparison tool (ACT); each panel shows the alignment of each reassembled genome against the reference sequence. (A) Populations 1 to 3. (B) Populations 4 to 6. (C) Populations 7 to 10. (D) Populations 10 to 12. S10 relocations from its original position can be detected as a yellow exchange within chromosome. Download FIG S4, PDF file, 0.10 MB. Copyright © 2023 Larotonda et al. 2023 Larotonda et al. https://creativecommons.org/licenses/by/4.0/ This content is distributed under the terms of the Creative Commons Attribution 4.0 International license.

10.1128/mbio.03432-22.5 FIG S5 Genome large rearrangement analysis in single clones isolated throughout the experiment. The NGS data each single clone was recovered and analyzed as described under Materials and Methods. The individuals isolated are described in Table S1. Each clone name is colored according to the population from it was isolated: P1 to P3, violet; P7 to P9, red; and P10 to P12 blue. Visualization was done using ACT; each panel shows the alignment of each reassembled genome against the reference sequence. S10 relocation from its original location can be noticed as in previous figures. Download FIG S5, PDF file, 0.08 MB. Copyright © 2023 Larotonda et al. 2023 Larotonda et al. https://creativecommons.org/licenses/by/4.0/ This content is distributed under the terms of the Creative Commons Attribution 4.0 International license.

10.1128/mbio.03432-22.9 DATA SET S1 **A** Mutations found at G250 in the 12 populations and the founding strains. The table shows the chromosomal location, the coordinates within the DNA molecule, the DNA change, the coverage (total number of reads) and the frequency within the reads (%), the codon change, the amino acid change, the affected gene, and the number of populations presenting the same DNA change for each mutation found. **B** Mutations found at G1000 across the 12 populations and the founding strains. The table shows the chromosomal location, the coordinates within the DNA molecule, the DNA change, the coverage (total number of reads) and the frequency within the reads (%), the codon change, the amino acid change, the affected gene, and the number of populations presenting the same DNA change for each mutation found. **C** Mutations found at G1000 in isolated smooth (A) or rugose (B) clones and the founding strains. The table shows the chromosomal location, the coordinates within the DNA molecule, the DNA change, the coverage (total number of reads) and the frequency within the reads (%), the codon change, the amino acid change, and the affected gene for each mutation found. **D** Mutations found at G250 in the FG clones and the founding strains. The table shows the chromosomal location, the coordinates within the DNA molecule, the DNA change, the coverage (total number of reads) and the frequency within the reads (%), the codon change, the amino acid change, the affected gene, and the number of populations presenting the same DNA change for each mutation found. Download Data Set S1, XLSX file, 0.1 MB. Copyright © 2023 Larotonda et al. 2023 Larotonda et al. https://creativecommons.org/licenses/by/4.0/ This content is distributed under the terms of the Creative Commons Attribution 4.0 International license.

As a general scenario, we found that at G250 mutations occur several times independently, suggesting a positive selection of most of them rather than genetic drift. In particular, we observed that a majority of the populations displayed mutations at the main flagellar regulators (Data Set S1A; Table 1).

### Evolutionary forces change beyond G250: adaptative, nonadaptative mutations, and hypermutators after 1,000 generations of experimental evolution.

We speculated that the mutations observed at G1000 may reflect those that are fixed in the long run, since after ~G500, biofilm formation decreases and growth rate remains roughly constant. After 1,000 generations of experimental evolution, we observed 512 fixed changes in at least 25% of the population at 396 sites of the genome of V. cholerae (Fig. 4A, right bars). In contrast to the G250 scenario, few mutational events occurred in more than one population (1.3 events per population on average). We observed a lower proportion of mutated pseudogenes (4.5% in G1000 versus 26.6% in G250) and an increase in the proportion of synonymous mutations (4.4% versus 19.4%) (Fig. 4B). We also noticed mutational events at several genes involved in DNA repair, such as *mutT* (VC1342), *recBCD* (VC2319-VC2320), *mutS* (VC0535), and *recJ* (VC2417). In particular, *mutL* (VC0345) was mutated in 8 of 12 populations (P1, P2, P3, P4, P5, P6, P7, and P11). Populations with intact DNA repair mechanisms (i.e., P8, P10, and P12) were those showing the lowest number of mutations (Fig. 4A). These data suggest that in most populations at G1000, many of the identified mutations may result from a hypermutator phenotype rather than selection. In all populations in which S10 is close to *ori1* (P1 to P6), we detected one or more mutations in DNA repair systems (Fig. 4A, blue). In contrast, half of the populations bearing S10 at an *ori1* distal position (P7 to P12) displayed an intact DNA repair pathway (Fig. 4A, red).

Although not the most frequent scenario, we also detected many DNA changes in the same gene or operon occurring many times independently or emerging in parallel in more than one population, likely indicating positive fitness selection throughout the evolution experiment (Table 2). On top of *aroG*, *ompT*, *flrABC*, and *tagE2* already acquired at G250, we found many mutations in interlinked cellular processes (38, 43, 44) such as rugosity, biofilm structure, and regulation (*vpsA*, *vpsB*, *vpsP*, *vpsR*, *capK*, and *rbmB*) (65–68), quorum sensing (*luxP*, *luxO*, and *luxU*) (69), flagellum biosynthesis (*flgA*, *flgI*, *flgK*, *fliG*, *fliO*, *flag*, and *flhA*) (39), and chemotaxis (*cheY* and *cheR2*) (39, 70). We also identified several altered functions such as extracellular macromolecular structures linked to virulence (toxin coregulated pilus [*tcp*], mannose-sensitive hemagglutinin [MSHA] pilus, type 6 secretion system [T6SS]), iron incorporation and metabolism (particularly vibriobactin pathway genes and *iscR*), lipopolysaccharide biosynthesis (*rfbA*, *rfbB*, *rfbV*, and *pgi*), and phosphate sensing and incorporation (*phoR*, *phoU*, and *phoH*). Interestingly, the latter ones have been linked to biofilm downregulation (71). Most of them were nonsynonymous or led to frameshifts likely to be detrimental for their biological function (Data Set S1B). We interpret these mutations in light of a change from a host-environmental life cycle to the relatively constant culture condition of the evolution experiment. Functions such as virulence, swimming, chemotaxis, pili production, T6SS, and micronutrient scavenging are energetically costly structures under strong selection in V. cholerae natural environment that can become a burden during *in vitro* evolution.

**TABLE 2:** Mutations occurring in multiple populations of evolved V. cholerae populations after 1,000 generations^*a*^

Gene/ORF	Function	Populations	Type of mutation
*rfbB* (VC0242)	Phosphomannose mutase	2, 3	NS
*rfbV* (VC0259)	Lipopolysaccharide biosynthesis	2, 5	NS
*manA-1* (VC0269)	Lipopolysaccharide biosynthesis mannose-6-phosphate isomerase	1, 3, 4, 6, 7, 8, 9, 11	FS
Intergenic (VC0341[*orn*], tRNAGly)	Unknown	1, 6	
*mutL* (VC0345)	Mismatch repair	1, 3, 4, 5, 6, 7, 11	NS, FS
*mshH*(VC0398)	MSHA biogenesis	1, 9	NS
Intergenic tRNA33-VC0449	Unknown	1, 5, 8	
Intergenic VC0511-12	Unknown	4, 7, 8, 9, 12	
*mutS* (VC0535)	Mismatch repair	2, 4	FS
VC0590	Permease	7, 11	NS
VC0603	Unknown (PNPLA domain-containing protein)	1, 5, 6, 7, 11	FS
*iscR* (VC0747)	Fe-S cluster sensor status and biogenesis	1, 3, 4, 5, 6, 9, 10, 11	NS, S, FS
*tcpC* (VC0831)	Toxin coregulated pilus	1, 3, 6, 7, 9	FS
*gcvA* (VC0896)	Transcriptional activation of the glycine cleavage system.	4, 9	NS
*vpsA* (VC0917)	Biofilm formation	2, 6	NS
*luxO* (VC1021)	Quorum-sensing regulation, biofilm formation	1, 2, 3, 5, 6, 10	NS
VC1724	Hypothetical protein	5, 6	FS
VC1761	Hypothetical protein (VPI-2)	4, 9	NS
*ompT* (VC1854)	Main porin	11, 12	NS, S, FS
*flhA*(VC2069)	Flagellar biosynthesis protein	1, 6	FS, NS
VC2105	Putative thioesterase	1, 4, 5, 7, 9, 12	NS
*flrA*	Flagellar master regulator	1, 2, 3, 4, 5, 6, 7, 9, 10, 11, 12	FS, NS
Intergenic (VC2286-VC2887)	Unknown	2, 3, 9	
Intergenic (VC2387-2388)	Unknown	1, 6	
*rimO* (VC2620)	Ribosome biogenesis	1, 6, 7	FS, NS
VC2684	Bifunctional aspartokinase/homoserine dehydrogenase	7, 10	NS
VC2707	Unknown	1, 9, 12	NS
VCA0044 (pseudogene)	Diguanylate cyclase	8, 12	NS
VCA0088 (pseudogene)	Proton/glutamate symporter	6, 7, 12	NS
VCA0168 (pseudogene)	BatD family protein	1, 2, 4, 6, 7, 8, 9, 11, 12	
VCA0354 (pseudogene)	DedA family protein	6, 9, 12	
Intergenic (VCA0591-VCA0592)	Unknown	6, 9	
*luxP* (VCA0737)	Quorum sensing	3, 7, 9	NS
VCA1031 (pseudogene)	Chemotaxis-like protein (10.1016/j.femsle.2004.08.039)	2, 4, 5, 6, 7, 8, 9, 11, 12	
*tagE2* (VCA1043)	Endopeptidase	1, 4, 5, 7, 8, 9, 12	NS, FS
intergenic (VCA1052-3)	Unknown	1, 3, 4, 5, 7	
*aroG* (VCA1036)	Aromatic amino acid synthesis	5, 7	NS
VCA1080	HlyD family secretion protein	1, 2, 6, 8	NS

a All mutations recovered and the details are described in Data Set S1B. We included intergenic regions specifying the flanking genes. FS, frame-shift mutation; MSHA, mannose-sensitive hemagglutinin; PNPLA, Patatin Like Phospholipase; NS, nonsynonymous mutation; ORF, open reading frame; S, synonymous mutation.

### Mutations linked to rugose phenotype.

Throughout the experimental evolution, we observed the emergence of biofilms and aggregates linked to a rugose phenotype described earlier (Fig. 2; Fig. S1 and S2). To uncover genomic changes associated with biofilm emergence, we fully sequenced the genome of a rugose and a smooth clone from a fast-growing (P1) and a slow-growing population (P12). After 1,000 generations, the clones originated from P1 and P12 fixed 9 and 6 mutations, respectively (Fig. 4C), independently of their smooth (S) or rugose (R) phenotype (1 mutation fixed every 133 ± 30 generations). Although some DNA changes were common among R and S colonies (Data Set S1C), we found some differential mutations potentially involved in biofilm and rugose phenotype emergence (Table 3). In particular, mutations in *vpsR*, *vpsB*, *cdgB*, and VCA1031 were previously linked to rugose phenotype by Yildiz and coworkers (46, 72, 73). *vpsR* is a positive regulator of the *vps cluster* that suffered a nonsynonymous mutation (D40Y) in the S colony derived from P1. The *vpsB* gene codes for an enzyme that participates in the synthesis of nucleotide sugar precursor necessary for polysaccharide biosynthesis. We detected a premature stop codon in the S colony collected from P12 (P12S). The P12R displayed a nonsynonymous mutation (D101G) at the *cdgB*, a GGDEF protein upregulated in rugose clones (73, 74). It also showed a nonsynonymous mutation in the *iscR* gene that codes for a transcription factor regulating genes related to iron starvation, oxidative stress, and oxygen limitation in Escherichia coli and linked to virulence in other bacteria (75). Meanwhile, the P1S colony displayed a mutation at VCA1031, encoding a putative methyl-accepting chemotaxis protein previously reported to be overexpressed in rugose strains having a genetic *ΔcdgC* background (46). In parallel, P1R showed an intergenic mutation also found at G250, at G1000, and on fast-growing clones (see above). Finally, P12S displayed a synonymous mutation in the *rpoA* gene, a main component of the RNA polymerase. We found two other differential mutations unlikely to be causal rugose phenotypes but that might have been coselected. In summary, the S and R clones showed alterations in genes previously linked to biofilm generation and c-di-GMP regulation. However, we detected other changes in R clones such as in *iscR* and at the intergenic region between VC0448 and VC0449.

**TABLE 3:** Mutations linked to rugose phenotype on V. cholerae populations evolved for 1,000 generations^*a*^

Gene	Function	Population	Type of mutation
*vpsR* (VC0665)	Positive biofilm regulator	1S	NS
*vpsB* (VC0918)	Biofilm	12S	NS
*cdgB* (VC1029)	GGDEF protein	12R	NS
VCA1031 (pseudogene)	Chemotaxis-like protein	1S	NS
*iscR* (VC0747)	Fe-S cluster sensor status and biogenesis	12R	NS
*rpoA* (VC2571)	α-Subunit of RNA polymerase	12S	S
Intergenic (VC0341[*orn*], tRNA-Gly)	Unknown	1R	

a Data Set S1C includes the full list of mutations and their details. FS, frame-shift mutation; NS, nonsynonymous mutation; S, synonymous mutation.

### An increase in growth rate is often associated with motility loss.

**FIG 5:**
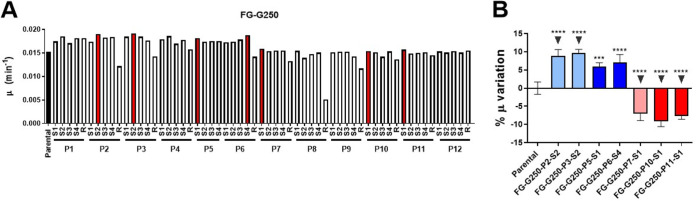
Specific mutations associated with high growth rate. (A) Screening fast-growing clones: five isolated clones per population (four S and one R) were tested per triplicate. The graph shows the average μ of each clones compared to the parental strain (black). Clones indicated in red were selected for further study. (B) The clones selected in panel A were subjected to growth curves by triplicate, and the percentage of variation of μ with respect to the parental strain is shown (*n* = 4). The clones indicated with an arrow were subjected to deep sequencing. Statistical significance was analyzed by one-way ANOVA two-tailed test. Then, Tukey’s test was done to compare the mean values obtained for each strain. Statistically different means are indicated as follows: ***, *P* < 0.001; ****, *P* < 0.0001.

To uncover mutations associated with fast growth, we isolated four smooth colonies and a rugose clone (S1 to S4 and R, respectively) from P1 to P12 at G250, the moment in experimental evolution when we noticed a sudden growth rate increase in all populations. Next, we performed automated growth curves of these clones using as a reference the parental strain (Table S1). We calculated the growth rate for each strain (Fig. 5A), and we selected the fastest growing clones, from P1 to P6 and from P7 to P12. We called these clones fast-growing clones followed by population of origin and its smooth or rugose status. Thus, we isolated the clones FG-2-S2, FG-3-S2, FG-7-S1, FG-10-S1, and FG-11-S1. Interestingly, the three former clones grew 7% to 10% faster than the parental strain. Meanwhile, FG-G250-7-S1, FG-G250-10-S1, and FG-G250-11-S1 improved their growth rate compared to their ancestors; they still displayed μ reductions of ~7% with respect to the parental strain (Fig. 5B). This is another line of evidence indicating that S10 genomic location cannot be suppressed in strains in which this locus is located far from the replication origin.

To identify the mutations responsible of μ increase, we fully sequenced FG-G250-2-S2, FG-G250-3-S2, FG-G250-7-S1, FG-G250-10-S1, and FG-G250-11-S1 genomes and compared them to the ancestral strains. Overall, strains displayed 1 to 3 changes in the DNA sequence (1 mutation fixed every ~140 generations) (Fig. 4C). In the 5 strains, we found 8 different mutations (Table 4). All isolated strains harbored alterations either at *flrA* (4 of 5) or at *flrB* (1 strain), the main flagellum regulators, which were previously observed at the population level at G250 (Table 1). Three other mutations accompanied the latter: a nonsilent mutation at a magnesium transporter (*mgtE*), a point mutation at an intergenic base between genes VC0448 and VC0449, and a frameshift mutation at *ompT*. Except for the frameshift mutation, we found the exact same mutations at G250 and most of them at G1000 (Data Set S1D). Analysis of gene order among the FG clones showed that genome structure did not suffer large inversions or translocations affecting S10 genomic location (Fig. S5). Therefore, strains in which this locus is far from *ori1* did not suppress the phenotype through large chromosomal rearrangements.

**TABLE 4:** Mutations observed in fast-growing clones isolated from G250^*a*^

Strain	Gene	Reference	Mutation	Result
FG-G250-2	*flrA* (VC2137)	A	G	I303T
FG-G250-2	Intergenic space (tRNA33-VC0449)	G	T	ND
FG-G250-3	*flrB* (VC2136)	TGTGAGCGAGTGGTGAA	T	FS(294)
FG-G250-7	*flrA* (VC2137)	AC	A	FS(461)
FG-G250-10	*flrA* (VC2137)	A	AC	FS(461)
FG-G250-10	*mgtE* (VCA0818)	G	C	G430R
FG-G250-11	*flrA* (VC2137)	A	C	L356R

a The details are described in Data Set S1D. FS, frame-shift mutation; ND, not determined.

**FIG 6:**
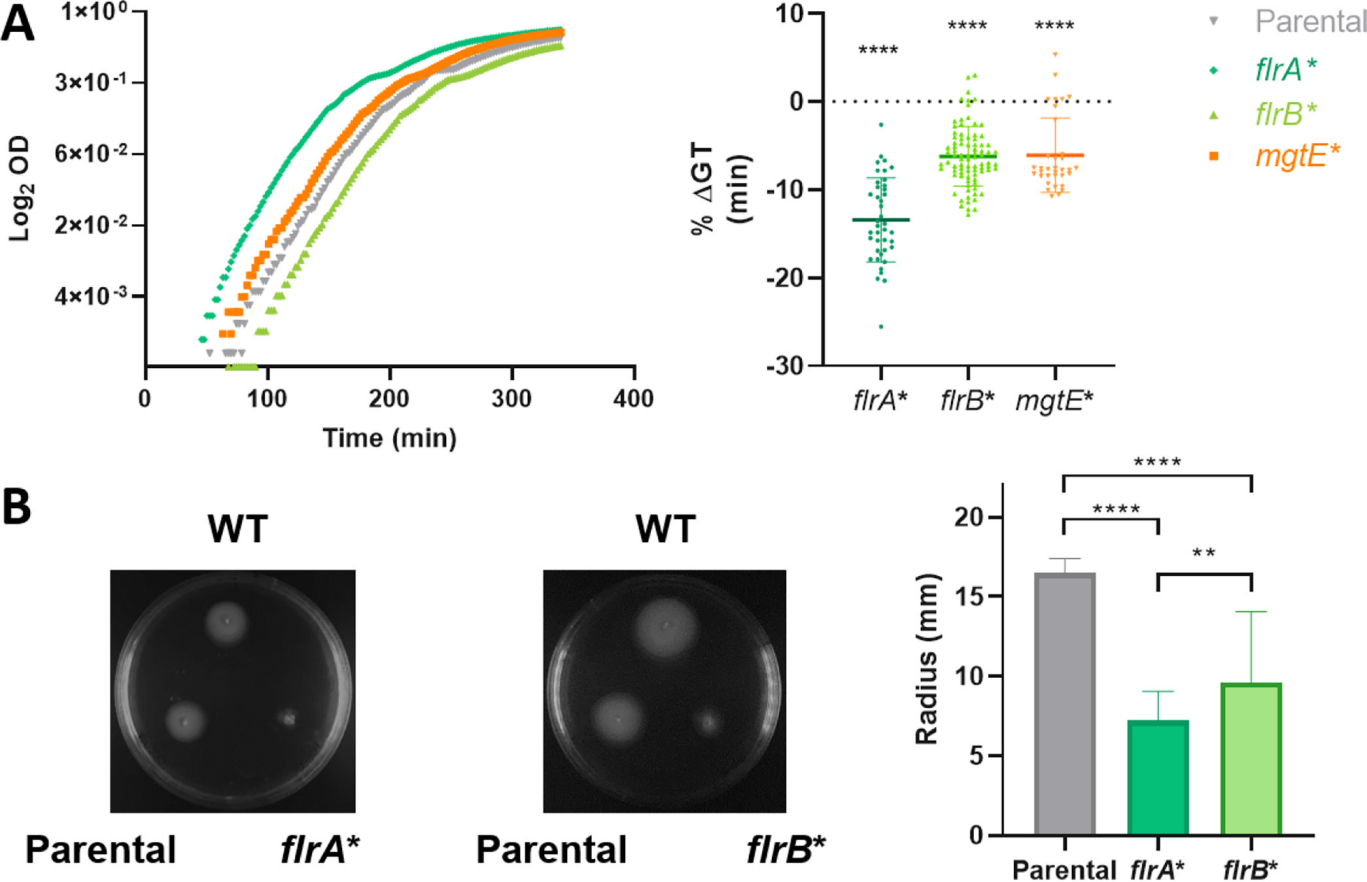
Fast growth-associated mutations increase growth rate in a V. cholerae naive context. (A) Mutations associated with fast growth (Table 4) were introduced into wild-type V. cholerae using MuGENT. The growth rate of these mutants was determined in automated growth curves performed by triplicate (*n* = 11). The left panel shows a representative growth curve. The right panel represents the median ± CI of the percentage of generation time (GT) variation of the indicated mutants with respect to the wild-type strain (WT). Statistical significance was determined using one-way ANOVA. Then, Dunnet’s test was applied for multiple comparisons. (B) Mutations in *flrA* and *flrB* reducing generation time reduce V. cholerae swimming capacity. Left panel show a representative swimming assay of the WT, the parental strain bearing the selection cassette (parental), and the clone bearing the indicated mutation (flrA* and flrB*). The right panel shows the analyzed data for all the assays performed per triplicate (*n* = 9). Statistical significance was analyzed by one-way ANOVA. Then, Tukey’s test was done to compare the mean values. **, *P* < 0.01; ****, *P* < 0.0001. OD, optical density.

To demonstrate the role of mutations in *flrA*, *flrB*, and *mgtE* on growth rate enhancement, we performed Multiplex Genome Editing by Natural Transformation (MuGENT) (76, 77) on naive V. cholerae N16961 to introduce point mutations, small indels, or short insertions. *flrA* is higher in the hierarchy of flagellum regulation (39) and is more prevalent on FG strains. FlrA is a 488-amino acid-long protein bearing an REC domain, a central ATPase associated with diverse cellular activities (AAA+), and a C-terminal helix-turn-helix domain responsible for DNA binding (78). The *flrA* frameshifts observed on populations and on in the FG-G250 clones occurred exclusively at the end of the latter domain, on positions 418 to 488 of the protein. The nonsynonymous mutations occurred at the AAA+ domain (positions 122 to 417 of FlrA). Using a V. cholerae wild-type background, we generated a frameshift at the same in *flrA* position (Table S2), obtaining several independent mutant clones that we tested on automated growth curves (Fig. 6A). This mutation increased the growth rate with respect to the wild-type strain. Similarly, the introduction of a frameshift in *flrB* resulted in a growth rate improvement. Also, the introduction of a nonsynonymous mutation at *mgtE* displayed a similar phenotype (Fig. 6A). To quantify the effect of individual mutations, we determined the percentage of generation time variation (%GT). Mutants bearing the *flrA* frameshift displayed the strongest reduction with a 13.41% ± 4.7% reduction of their generation time (95% CI, 14.9 to 11.9; *P* < 0.0001). Meanwhile, *flrB* and *mgtE* mutants displayed a 6% generation time reduction (95% CI, 6.9 to 5.4 and 7.5 to 4.6, respectively; *P* < 0.0001). These results coincide with the observed increase in growth rate of FG clones. Simultaneously, frameshifts in *flrA* and *flrB* caused a reduction of motility as shown by swimming assays (Fig. 6B), demonstrating that growth rate increase is associated with the loss of *flrA* and *flrB* function.

10.1128/mbio.03432-22.7 TABLE S2 Full list of primers used in this study. Download Table S2, PDF file, 0.06 MB. Copyright © 2023 Larotonda et al. 2023 Larotonda et al. https://creativecommons.org/licenses/by/4.0/ This content is distributed under the terms of the Creative Commons Attribution 4.0 International license.

In summary, these results demonstrate that the mutations observed in the FG clones increased the growth rate in V. cholerae. They also suggest that some of the mutations detected at G250 and maybe at G1000 also contributed to the growth optimization of this microorganism.

## DISCUSSION

Increasing evidence suggests that gene order within the bacterial chromosome contributes to cellular homeostasis by coordinating key entangled processes such as chromosome structuration, cell cycle, replication, and the expression of genetic information. While genome reshuffling experiments have not been yet performed in bacterial systems (79), approaches such as comparative genomics (17, 18, 21, 23, 31, 22), systems biology (20), large DNA inversions (80–82), and relocation of individual gene sets (16, 19, 83–85) have provided support to this notion. Genes encoding the genetic information flow are interesting models to test the role of genomic position on cellular physiology. In this work, we took this “positional genetics” approach (in which genes of interest are systematically relocated within the genome) a step further, by observing how movants bearing S10 at different genomic locations evolve *in vitro* for 1,000 generations in fast-growing conditions, those in which phenotypic effects were stronger (Fig. 1).

Pioneer work by Richard Lenski and coworkers on long-term evolution was done for more than 75,000 generations (and counting!) in E. coli. However, the strongest fitness improvement occurred during the first 2,000 generations (58, 60). Therefore, although we performed a shorter experiment, we believe that we observed a representative part of the evolutionary trajectory. Meanwhile, we developed a different approach: we used rich medium and a model organism that grows almost twice as fast as E. coli. Also, our system used 1 log larger bottlenecks, improving the chance of mutant selection and lowering the effects of genetic drift (i.e., we transferred 10^9^ instead of 10^8^ CFU). Finally, we evolved populations bearing the key locus S10 at different genomic locations.

Along with this experiment, we found the emergence of abundant biofilms and aggregates in all populations. Other experimental evolution approaches also led to biofilms and/or aggregates. In Acinetobacter baylyii ADP1 (86), it was the consequence of insertion sequences (IS) action that led to evolution of aggregating clones. The studies on Burkholderia cenocepacia (87) and Bacillus subtilis (88) actively selected for biofilm emergence with a wide variety of clones with clear fitness advantages such as labor division and heterogenicity emergence within the structure. A limitation of our study is that we sequenced rugose clones at G1000 instead of G250, when most biofilms were observed. At this time point, many other unrelated mutations could be coselected with the DNA changes causing rugose phenotypes. We described biofilm emergence throughout experimental evolution, and we observed mutations co-occurring with this phenotype that are in agreement with previous papers (38, 66–68, 72, 74).

During the experiment, the growth rate improved for all V. cholerae populations. This trait was not initially noticed in the long-term evolution experiment performed in E. coli, although fitness and cell size increment were observed since the initial steps of the experiment (60, 89, 90). Recently, an increase in growth rate could also be detected (91) in line with “bacterial growth laws” that correlate growth rate to cell size (92). In V. cholerae experimental evolution, growth rate improvement was readily detected. This could be the result of employing a richer broth, the evolution strategy, and/or the faster growth nature of V. cholerae. Since growth rate is a good proxy for fitness (50, 93, 94), we propose that the increment in growth rate observed reflects, at least in part, the expected fitness improvement. The growth stabilization after G500 could be the result of high divergence between populations or the consequence of terminating the experiment relatively early. Further generations of evolution should lead to growth increases in a stepwise manner.

Deep sequencing of the ancestral clones, the populations at G250, and at the end of the experiment suggested different selection forces at each time point. At G250, positive selection seems to be the main force driving mutation. This scenario changes at G1000, when most of them seemed not to be under strong selection. This is reinforced by the fact that many populations showed inactivation of DNA repair systems that in turn resulted on average in more DNA changes (Fig. 4A). Interestingly, populations where S10 is close to *ori1* (P1-6) tend to have more alterations in repair systems and concurrently, more mutations at G1000.

Some DNA changes arose in more than one flask, in particular inactivating genes encoding functions that might become unnecessarily costly during *in vitro* continuous culture such as virulence, motility, and nutrient scavenging. Notably, many functions associated with virulence were found inactivated (MSHA, *tcp*, T6SS, etc.) throughout the evolution experiment. We also noticed several mutations at quorum-sensing (QS) and chemotaxis-related genes at G1000 (see above). The former seems to be a common feature of laboratory domesticated V. cholerae strains (95). Both chemotaxis and QS are important factors during host infection (96, 97). Taken together, these observations suggest that these mutations are likely to reduce V. cholerae virulence. Meanwhile, the increased growth and the higher biofilm-forming capacity may increase their infection capacity. Therefore, possible virulence reduction of the strains throughout experimental evolution is worth of investigation. In that case, understanding how resilient their encoding genes are in the absence of selective forces maintaining virulence should be studied.

Interestingly, mutations acquired at G250 were not lost, suggesting their continuous selection throughout the experiment. Moreover, in the case of flagellum biosynthetic pathway, we found, on top of those on the master regulators *flrAB*, mutations in transcriptional regulators of lower hierarchy such as *flhA* or components of the machinery such as *flgA*, *flgI*, or *flgK*.

At both G250 and G1000, we detected many mutations occurring in *a priori* noncoding regions such as intergenic regions and pseudogenes. We cannot discard biological effects interfering with promoter or regulatory regions, potential uncharacterized small RNAs, or pseudogenes expressing truncated but functional proteins. This might be the case with those occurring in several populations independently, such as the point mutations occurring at VCA0168, a pseudogene located on the secondary chromosome, mutated in 9 of 12 populations (Data Set S1).

Interestingly, S10 relocation to chromosome termini leads to a relatively strong fitness loss that is easily outcompeted by cells having an *ori1*-proximal location for the main ribosomal protein locus (50). This contributes to explain the S10 proximity to *ori1* conserved among *Vibronaceae* (49). However, this change in chromosomal architecture is not lethal nor does it result in cells with lower viability or a sick phenotype. An easy way to regain fitness upon S10 relocation to the *ter* region would be large-scale chromosomal rearrangements such as inversions, translocations, or S10 duplication. However, we did not observe them in the populations (Fig. S4) nor in the isolated clones sequenced (Fig. S5). Indeed, these rearrangements were a common feature of long-term evolved E. coli populations. In some cases, such as the ribose utilization operon, they were linked to a fitness gain (98). However, reverting S10 *ori1*-distal location could disturb the conserved structure of the bacterial chromosome. For example, essential genes are usually found in the replication leading strand to avoid deleterious conflicts between replication and transcription (7, 99). Large chromosome inversions able to relocate S10 close to *ori1* would encompass many highly transcribed essential genes that would not be viable or would be rapidly purified from populations. Similarly, large inversions or translocations could lead to replichore length imbalance. Meanwhile, chromosomal rearrangements were possible since we detected some events in sequenced clones (e.g., a large translocation in G250-FG-2; Fig. S5). However, these alterations are unlikely to be associated with the observed phenotypes (a faster growth), since reconstructing point mutations in flagellum regulators and in the *mgtE* gene fully reproduced the phenotype in a wild-type context. Also, point mutations suppressing S10 location change were not detected in our experiment after 1,000 generations. Therefore, we think that suppressing S10 either by altering chromosome architecture or by suppressor mutations is hard to achieve at least in this range of time. Meanwhile, under circumstances under which S10 *oriC*-proximal location becomes essential, these changes might be able to emerge. For instance, during infections in the Drosophila melanogaster model (96, 97, 100), S10 relocation to chromosomal termini leads to reduction of CFU/fly by orders of magnitude (49). Thus, experimental evolution in the fly gut or in conditions mimicking that of the strains S10Tnp-1120 and S10TnpC2 + 479 may lead to large chromosomal rearrangements or suppressor mutations that counterbalance S10 relocation far from *ori1*.

Importantly, we were able to distinguish mutations that improved bacterial growth from those with increasing biofilm-forming capacity. In particular, mutations inactivating the main flagellum regulators emerged in 7 and 11 of 12 populations at G250 and G1000, respectively. Their reintroduction into the wild-type context increased V. cholerae growth, demonstrating its specific effect and indicating that the rest of the detected mutations contributed growth enhancement. *flrAB* mutations also caused a reduction in motility that provide an ecological and physiological interpretation under the light of recent studies (101–103). These works show a trade-off between motility/chemotaxis and growth rate. In changing environmental conditions, cells deal with several simultaneous energetically costly processes such as growth, motility chemotaxis, and metabolism. Energy allocation to flagellar genes production, rotation of the flagellum, and, to a lesser extent, chemotaxis significantly reduces growth in E. coli (102, 103). It is advantageous to dedicate energy to motility and chemotaxis in poor and heterogenous environments. Consistently, at G1000, we also noticed many mutations related to other swimming and chemotaxis genes (e.g., *fliO*, *flrC*, *cheY*, *cheR2*, *flgA*). In summary, many of the mutations detected throughout our experiment altered the motility/growth interplay in all the studied populations. However, many other mutations that contribute to improving fitness remain to be better characterized. For instance, mutations on *mgtE*, which are not clearly related to motility, also improved the growth rate. This opens the possibility of finding other mutations on genes not related to motility capable of improving the growth of an already very fast growth bacterium. In particular, magnesium might be a limiting micronutrient in fast-growing conditions, a factor to take into account when optimizing bacterial growth. Such a factor is worth testing in the V. cholerae relative Vibrio natriegens (GT = 10 min), since at the time it is a biotechnologically useful organism and would help to further push the growth on the fastest growing known organism.

After 1,000 generations of experimental evolution, although all populations shortened their generation time, those harboring S10 locus close to *ori1* remained the fastest. This evidences the great potential of positional genetics approach (49) as a synthetic biology tool to condition cell physiology to permanently avoid the escape of suppressor variants. The latter conditions the design of stable genetic circuits. For instance, recent work by 1Izard et al. (104) created a growth switch in E. coli by controlling RNA polymerase expression from an inducible promoter that required three copies of the main repressor to avoid regulation escape. Despite the powerful nature of this synthetic circuit, escape could still be detected. Positional genetics offers a new way to cope with this problem to generate robust genetic circuits immune to suppressor mutations.

### Conclusions.

By applying the positional genetics approach to ribosomal protein genes, we contributed to understanding the role of gene order in bacterial cell physiology. The present work incorporates the evolutionary aspect of studies on positional genetics, expanding our knowledge on the long-term implication of gene order on genome evolution. The role of the genomic position of other key genes remains to be explored, such as ribosomal RNAs (105), tRNA^UBI^ (22, 24), RNA polymerase (51), and ATP synthase (17), among others. This is central to understanding how the genome primary structure affects cell physiology, in particular growth rate and genome evolution. Such knowledge will enable bacterial growth repurposing while allowing understanding of the behavior of more complex biological systems, thus promising a deep impact in genome design, bioengineering, and biotechnology.

## MATERIALS AND METHODS

### Bacterial strains and plasmids and culture conditions.

All strains were derived from V. cholerae serotype O1 biotype El Tor strain N16961 with Str^R^ (106). Bacterial growth was done in lysogeny broth (LB) at 37°C agitating at 200 rpm. The strains and plasmids used in this study are listed in Table S1.

### General procedures.

Genomic DNA was extracted using the GeneJET genomic DNA purification kit, while plasmid DNA was extracted using the GeneJET plasmid miniprep kit (Thermo Scientific) or ADN Puriprep B and P (InbioHighway, Argentina). PCR assays were performed using DreamTaq, Phusion High-Fidelity PCR Master Mix (Thermo Scientific), or MINT 2X (InbioHighway, Argentina). The oligonucleotides described in Table S2 were purchased from Macrogen (Seoul, Korea).

### Experimental evolution setting.

We studied four different strains bearing the S10 locus either close to their wild-type location (Par-1120 and S10Tnp-35; Table S1) or far from *ori1* (S10Tnp-1120 and S10TnpC2 + 479; Table S1). Overnight cultures of each strain were used to obtain isolated colonies in LB agar Str plates. For each one, we picked three different individual colonies to start 5-mL independent cultures on LB Str that were cultured overnight at 37°C agitating at 180 rpm. Then, these cultures were frozen at −80°C on LB 10% dimethyl sulfoxide (DMSO; Merck). These stocks constitute the starting point of the experiment. The first inoculation was done with 50 μL of each initial culture (10e9 CFU/mL as assessed by plating) in 250 mL and cultured overnight (ca. 14 generations). Then, each population was propagated in 250-mL (7 generations) or 500-mL (8 generations) Erlenmeyer flasks. Throughout the experiment, the cells were cultured at 37°C agitating at 200 rpm on a dry incubator (Infors HT). For each passage, 1 mL of the previous flask is transferred to the next step. When clumps or biofilms appeared, we did not let the culture still for long time to avoid counterselection of aggregates and biofilms. Also, we used 1-mL pipette tips that allowed us to pipet up and down several times in case cell clumps were too big to entirely pass through the tip. Populations were regularly checked for contamination by plating in LB Str. Colonies with unusual morphologies were checked phenotypically and by PCR amplifying the V. cholerae specific gene *rctB* (3,398 to 3,407; Table S2) or a specific region of the secondary chromosome (Tgt4_1 to Tgt4_4; Table S2). During the first 400 generations, the populations were frozen by centrifuging 10 mL of each culture in 14-mL Falcon tubes at 5,000 rpm for 7 to 10 min, discarding the supernatant, and resuspending the cells on 1 mL of LB supplemented with 10% of DMSO (Merck) frozen at −80°C until later use. After G400, the populations were frozen every 100 generations. Then, the cultures were frozen at −80°C until later use.

### Automated growth curve measurements.

Overnight cultures of the indicated microorganism were diluted 1/1,000 in LB. Bacterial preparations were distributed by triplicate for evolved populations and sextuplicate for the ancestral clones in 96-well microplates. Growth curve experiments were performed using a TECAN Infinite Sunrise microplate reader, with absorbance (620 nm) taken at 5-min intervals for 5 h with agitation at 37°C. The experiments were done at least twice independently. The slopes during the exponential phase were directly obtained using a home-made Python script coupled to a growth rates program (73). To filter noise due to growth variations among experiments, the obtained μ was relativized to the ancestral clones, and the relativized values were multiplied by the average of the ancestral clones throughout all the experiments. This normalization did not affect the results since raw data provided a similar picture as depicted in Fig. S3D. The percentage of GT was calculated as follows: %GT = (GT/GT^WT^ − 1) × 100.

### Biofilm formation assays.

Nine-well PVC microtiter plates (BD Falcon) were used to monitor biofilm formation as described previously (107). Briefly, LB was inoculated with a 1/100 dilution directly obtained from the liquid overnight cultures of each population in rich medium. After inoculation, microtiter plates were incubated at 37°C for 24 h and rinsed, and 150 μL of a 0.1% solution of crystal violet was added to each well. The plates were incubated at room temperature for 30 min and rinsed, and biofilm formation was tested as follows: crystal violet was solubilized by addition of 150 μL of ethanol-acetone (80:20), and the OD_570_ was determined. The results are presented as the means of four replicate wells in three independent experiments.

### Deep sequencing analysis.

The quality of Illumina reads was visualized by FastQC v0.10.1 Babraham Bioinformatics (http://www.bioinformatics.babraham.ac.uk/projects/fastqc/). All reads of 32 samples were aligned against the two chromosomes of V. cholerae (GenBank NC_002505 and NC_002506, 17 December 2014), using paired end mapping mode of BWA “mem” v0.7.4 (108) with the option “−M.” The reference chromosome I was modified since the reference genome sequence (AE003852) showed an inversion around ori1 flanked by two rRNA operons (*rrnB* and *rrnG-H*) (48). Output SAM files were converted to BAM files using SAMtools v0.1.19 (109). The “Add Read Groups” step was made by Picard v1.131 (http://picard.sourceforge.net/). The aligned reads in BAM files were realigned with the command “IndelRealigner” implemented in GATK v2.2.16 (110). “MarkDuplicates” step from Picard flagged duplicates reads. We kept only uniquely mapped reads, using SAMtools (option “view –bq 20”). A summary of the sequencing depth for each population is summarized in Table S3. Then, mpileup files were generated using SAMtools without BAQ adjustments (option “mpileup –B”). SNPs and indels were called by the option “pileup2cns” of Varscan2 v2.3.2 (111) with a minimum depth of 10 reads, a threshold of 20 for minimum quality, a threshold of 0.25, and 0.8 for the minimum variant allele frequency for heterogeneous population strains and subclone isolated strains, respectively. The coverage around the mutation was checked with a Python script with a minimum depth of 7 reads. The annotation of identified variants was processed by SnpEff v4.1 (112). Common mutations between all sequenced strains were ignored, indicating differences with the reference sequence. We also looked at possible inversions or alterations in gene order in evolved populations. For this we employed Spades 3.6.2 (113) for assembly of trimmed reads with the kmer options “−k 21,51,71” and “−careful.” Contigs were ordered and oriented using CONTIGuator 2.7.4 (114) using the two chromosomes of V. cholera as references. The Artemis comparison tool (ACT) (115) was used for synteny visualization and for the search for dynamics of large rearrangements such as translocations and inversions. All data generated and analyzed during this study is included in this published article, its supplementary information files, and publicly available repositories. The Illumina Deep Sequencing data sets are deposited at the GenBank SRA as BioProject PRJNA816505.

### Multiplex Genome Editing by Natural Transformation (MuGENT).

To introduce the mutations observed throughout the evolution experiment, we employed MuGENT (76, 77). Briefly, the wild-type strain (Table S1) was grown overnight from a single colony at 37°C in LB with agitation. This overnight culture was diluted 10^−3^ into fresh medium, and the strain was grown to an OD_600_ ~0.5. The cells were washed by centrifugation and resuspended at the original volume in 1× instant ocean sea salts (7 g/liter) (Blacksburg) and autoclaved chitin powder (Sigma-Aldrich) and incubated overnight without agitation at 30°C to induce competence. In parallel, DNA fragments harboring the mutations were synthesized using PCR assembly, cloned using a pEASY-Blunt Zero Cloning kit (Transbiotech, China), and verified using Sanger sequencing (Macrogen). Next, we cotransformed the targeted loci containing the desired chromosomal alterations and a spectinomycin resistance cassette directed to a neutral locus at the intergenic space between VC1903 and VC1902 regions (116) using 3 μg of each DNA molecule. After overnight incubation at 30°C without shaking, the sample was vortexed and plated in LB supplemented with spectinomycin. Then, several colonies were screened and confirmed by MASC-PCR (77).

### Motility assay.

The effect of mutations in *flrA* and *flrB* on the motility of V. cholerae was tested on LB soft agar plates (0.3s% bacteriological agar). Next, 2 μL of bacterial suspension (OD_600_ = 1) was inoculated into the agar of each plate by triplicate to test bacterial swimming. The plates were incubated for 12 h at 37°C, and then the diameter of the motility halos was measured using ImageJ (117). The results are reported in mm of bacterial extension on the media.

10.1128/mbio.03432-22.8 TABLE S3 Sequence depth of strains and populations sequences during the experimental evolution of V. cholerae. We show for each population and each strain sequenced the total number of reads, the read length, and the coverage as the median of sequencing depth. The sequenced samples are colored according to their ancestral strain: parental + 1120 (purple), S10Tnp-35 (cyan), S10Tnp-1120 (red) and S10TnpC2 + 479 (blue). Download Table S3, PDF file, 0.09 MB. Copyright © 2023 Larotonda et al. 2023 Larotonda et al. https://creativecommons.org/licenses/by/4.0/ This content is distributed under the terms of the Creative Commons Attribution 4.0 International license.

## References

[1] Badrinarayanan A, Le TB, Laub MT. 2015. Bacterial chromosome organization and segregation. Annu Rev Cell Dev Biol 31:171–199. doi:10.1146/annurev-cellbio-100814-125211. 26566111 PMC4706359

[2] Brickner J. 2017. Genetic and epigenetic control of the spatial organization of the genome. Mol Biol Cell 28:364–369. doi:10.1091/mbc.e16-03-0149. 28137949 PMC5341720

[3] Misteli T. 2020. The self-organizing genome: principles of genome architecture and function. Cell 183:28–45. doi:10.1016/j.cell.2020.09.014. 32976797 PMC7541718

[4] Surovtsev IV, Jacobs-Wagner C. 2018. Subcellular organization: a critical feature of bacterial cell replication. Cell 172:1271–1293. doi:10.1016/j.cell.2018.01.014. 29522747 PMC5870143

[5] Lioy VS, Junier I, Boccard F. 2021. Multiscale dynamic structuring of bacterial chromosomes. Annu Rev Microbiol 75:541–561. doi:10.1146/annurev-micro-033021-113232. 34343019

[6] Touchon M, Rocha EP. 2016. Coevolution of the organization and structure of prokaryotic genomes. Cold Spring Harb Perspect Biol 8:a018168. doi:10.1101/cshperspect.a018168. 26729648 PMC4691797

[7] Rocha EP. 2008. The organization of the bacterial genome. Annu Rev Genet 42:211–233. doi:10.1146/annurev.genet.42.110807.091653. 18605898

[8] Le TB, Laub MT. 2014. New approaches to understanding the spatial organization of bacterial genomes. Curr Opin Microbiol 22:15–21. doi:10.1016/j.mib.2014.09.014. 25305533 PMC4258129

[9] Dorman CJ. 2013. Genome architecture and global gene regulation in bacteria: making progress towards a unified model? Nat Rev Microbiol 11:349–355. doi:10.1038/nrmicro3007. 23549066

[10] Le TB, Imakaev MV, Mirny LA, Laub MT. 2013. High-resolution mapping of the spatial organization of a bacterial chromosome. Science 342:731–734. doi:10.1126/science.1242059. 24158908 PMC3927313

[11] Le TB, Laub MT. 2016. Transcription rate and transcript length drive formation of chromosomal interaction domain boundaries. EMBO J 35:1582–1595. doi:10.15252/embj.201593561. 27288403 PMC4946140

[12] Lioy VS, Junier I, Lagage V, Vallet I, Boccard F. 2020. Distinct activities of bacterial condensins for chromosome management in *Pseudomonas aeruginosa*. Cell Rep 33:108344. doi:10.1016/j.celrep.2020.108344. 33147461

[13] Lioy VS, Cournac A, Marbouty M, Duigou S, Mozziconacci J, Espeli O, Boccard F, Koszul R. 2018. Multiscale structuring of the *E. coli* chromosome by nucleoid-associated and condensin proteins. Cell 172:771–783.e18. doi:10.1016/j.cell.2017.12.027. 29358050

[14] Meyer S, Reverchon S, Nasser W, Muskhelishvili G. 2018. Chromosomal organization of transcription: in a nutshell. Curr Genet 64:555–565. doi:10.1007/s00294-017-0785-5. 29184972

[15] Rocha EP. 2004. The replication-related organization of bacterial genomes. Microbiology 150:1609–1627. doi:10.1099/mic.0.26974-0. 15184548

[16] Slager J, Veening JW. 2016. Hard-wired control of bacterial processes by chromosomal gene location. Trends Microbiol 24:788–800. doi:10.1016/j.tim.2016.06.003. 27364121 PMC5034851

[17] Sobetzko P, Travers A, Muskhelishvili G. 2012. Gene order and chromosome dynamics coordinate spatiotemporal gene expression during the bacterial growth cycle. Proc Natl Acad Sci USA 109:E42–E50. doi:10.1073/pnas.1108229109. 22184251 PMC3258614

[18] Kosmidis K, Jablonski KP, Muskhelishvili G, Hutt MT. 2020. Chromosomal origin of replication coordinates logically distinct types of bacterial genetic regulation. NPJ Syst Biol Appl 6:5. doi:10.1038/s41540-020-0124-1. 32066730 PMC7026169

[19] Gerganova V, Maurer S, Stoliar L, Japaridze A, Dietler G, Nasser W, Kutateladze T, Travers A, Muskhelishvili G. 2015. Upstream binding of idling RNA polymerase modulates transcription initiation from a nearby promoter. J Biol Chem 290:8095–8109. doi:10.1074/jbc.M114.628131. 25648898 PMC4375467

[20] Yubero P, Poyatos JF. 2020. The impact of global transcriptional regulation on bacterial gene order. iScience 23:101029. doi:10.1016/j.isci.2020.101029. 32283521 PMC7155222

[21] Muskhelishvili G, Travers A. 2014. Order from the order: how a spatiotemporal genetic program is encoded in a 2-D genetic map of the bacterial chromosome. J Mol Microbiol Biotechnol 24:332–343. 25732336 10.1159/000368852

[22] Hu XP, Lercher MJ. 2021. An optimal growth law for RNA composition and its partial implementation through ribosomal and tRNA gene locations in bacterial genomes. PLoS Genet 17:e1009939. doi:10.1371/journal.pgen.1009939. 34843465 PMC8659690

[23] Sobetzko P, Glinkowska M, Travers A, Muskhelishvili G. 2013. DNA thermodynamic stability and supercoil dynamics determine the gene expression program during the bacterial growth cycle. Mol Biosyst 9:1643–1651. doi:10.1039/c3mb25515h. 23493878

[24] Couturier E, Rocha EP. 2006. Replication-associated gene dosage effects shape the genomes of fast-growing bacteria but only for transcription and translation genes. Mol Microbiol 59:1506–1518. doi:10.1111/j.1365-2958.2006.05046.x. 16468991

[25] Japaridze A, Yang W, Dekker C, Nasser W, Muskhelishvili G. 2021. DNA sequence-directed cooperation between nucleoid-associated proteins. iScience 24:102408. doi:10.1016/j.isci.2021.102408. 33997690 PMC8099737

[26] Muskhelishvili G, Sobetzko P, Mehandziska S, Travers A. 2021. Composition of transcription machinery and its crosstalk with nucleoid-associated proteins and global transcription factors. Biomolecules 11:924. doi:10.3390/biom11070924. 34206477 PMC8301835

[27] Block DH, Hussein R, Liang LW, Lim HN. 2012. Regulatory consequences of gene translocation in bacteria. Nucleic Acids Res 40:8979–8992. doi:10.1093/nar/gks694. 22833608 PMC3467084

[28] Scholz SA, Diao R, Wolfe MB, Fivenson EM, Lin XN, Freddolino PL. 2019. High-resolution mapping of the *Escherichia coli* chromosome reveals positions of high and low transcription. Cell Syst 8:212–225.e9. doi:10.1016/j.cels.2019.02.004. 30904377 PMC6508686

[29] Bryant JA, Sellars LE, Busby SJ, Lee DJ. 2014. Chromosome position effects on gene expression in *Escherichia coli* K-12. Nucleic Acids Res 42:11383–11392. doi:10.1093/nar/gku828. 25209233 PMC4191405

[30] Dryselius R, Izutsu K, Honda T, Iida T. 2008. Differential replication dynamics for large and small *Vibrio* chromosomes affect gene dosage, expression and location. BMC Genomics 9:559. doi:10.1186/1471-2164-9-559. 19032792 PMC2612033

[31] Sonnenberg CB, Kahlke T, Haugen P. 2020. Vibrionaceae core, shell and cloud genes are non-randomly distributed on Chr 1: an hypothesis that links the genomic location of genes with their intracellular placement. BMC Genomics 21:695. doi:10.1186/s12864-020-07117-5. 33023476 PMC7542380

[32] van Eijk E, Boekhoud IM, Kuijper EJ, Bos-Sanders I, Wright G, Smits WK. 2019. Genome location dictates the transcriptional response to PolC inhibition in *Clostridium difficile*. Antimicrob Agents Chemother 63:e01363-18. doi:10.1128/AAC.01363-18. 30455241 PMC6355584

[33] Gerganova V, Berger M, Zaldastanishvili E, Sobetzko P, Lafon C, Mourez M, Travers A, Muskhelishvili G. 2015. Chromosomal position shift of a regulatory gene alters the bacterial phenotype. Nucleic Acids Res 43:8215–8226. doi:10.1093/nar/gkv709. 26170236 PMC4751926

[34] Fitzgerald S, Dillon SC, Chao TC, Wiencko HL, Hokamp K, Cameron AD, Dorman CJ. 2015. Re-engineering cellular physiology by rewiring high-level global regulatory genes. Sci Rep 5:17653. doi:10.1038/srep17653. 26631971 PMC4668568

[35] Moffitt JR, Pandey S, Boettiger AN, Wang S, Zhuang X. 2016. Spatial organization shapes the turnover of a bacterial transcriptome. eLife 5:e13065. doi:10.7554/eLife.13065. 27198188 PMC4874777

[36] Charles RC, Ryan ET. 2011. Cholera in the 21st century. Curr Opin Infect Dis 24:472–477. doi:10.1097/QCO.0b013e32834a88af. 21799407

[37] Reen FJ, Almagro-Moreno S, Ussery D, Boyd EF. 2006. The genomic code: inferring *Vibrionaceae* niche specialization. Nat Rev Microbiol 4:697–704. doi:10.1038/nrmicro1476. 16894340

[38] Teschler JK, Zamorano-Sanchez D, Utada AS, Warner CJ, Wong GC, Linington RG, Yildiz FH. 2015. Living in the matrix: assembly and control of *Vibrio cholerae* biofilms. Nat Rev Microbiol 13:255–268. doi:10.1038/nrmicro3433. 25895940 PMC4437738

[39] Echazarreta MA, Klose KE. 2019. *Vibrio* flagellar synthesis. Front Cell Infect Microbiol 9:131. doi:10.3389/fcimb.2019.00131. 31119103 PMC6504787

[40] Silva AJ, Benitez JA. 2016. *Vibrio cholerae* biofilms and cholera pathogenesis. PLoS Negl Trop Dis 10:e0004330. doi:10.1371/journal.pntd.0004330. 26845681 PMC4741415

[41] Alam M, Sultana M, Nair GB, Siddique AK, Hasan NA, Sack RB, Sack DA, Ahmed KU, Sadique A, Watanabe H, Grim CJ, Huq A, Colwell RR. 2007. Viable but nonculturable *Vibrio cholerae* O1 in biofilms in the aquatic environment and their role in cholera transmission. Proc Natl Acad Sci USA 104:17801–17806. doi:10.1073/pnas.0705599104. 17968017 PMC2077051

[42] Faruque SM, Biswas K, Udden SM, Ahmad QS, Sack DA, Nair GB, Mekalanos JJ. 2006. Transmissibility of cholera: in vivo-formed biofilms and their relationship to infectivity and persistence in the environment. Proc Natl Acad Sci USA 103:6350–6355. doi:10.1073/pnas.0601277103. 16601099 PMC1458881

[43] Conner JG, Zamorano-Sanchez D, Park JH, Sondermann H, Yildiz FH. 2017. The ins and outs of cyclic di-GMP signaling in *Vibrio cholerae*. Curr Opin Microbiol 36:20–29. doi:10.1016/j.mib.2017.01.002. 28171809 PMC5534393

[44] Wu DC, Zamorano-Sanchez D, Pagliai FA, Park JH, Floyd KA, Lee CK, Kitts G, Rose CB, Bilotta EM, Wong GCL, Yildiz FH. 2020. Reciprocal c-di-GMP signaling: incomplete flagellum biogenesis triggers c-di-GMP signaling pathways that promote biofilm formation. PLoS Genet 16:e1008703. doi:10.1371/journal.pgen.1008703. 32176702 PMC7098655

[45] Floyd KA, Lee CK, Xian W, Nametalla M, Valentine A, Crair B, Zhu S, Hughes HQ, Chlebek JL, Wu DC, Hwan Park J, Farhat AM, Lomba CJ, Ellison CK, Brun YV, Campos-Gomez J, Dalia AB, Liu J, Biais N, Wong GCL, Yildiz FH. 2020. c-di-GMP modulates type IV MSHA pilus retraction and surface attachment in *Vibrio cholerae*. Nat Commun 11:1549. doi:10.1038/s41467-020-15331-8. 32214098 PMC7096442

[46] Lim B, Beyhan S, Yildiz FH. 2007. Regulation of *Vibrio* polysaccharide synthesis and virulence factor production by CdgC, a GGDEF-EAL domain protein, in *Vibrio cholerae*. J Bacteriol 189:717–729. doi:10.1128/JB.00834-06. 17122338 PMC1797307

[47] Val ME, Soler-Bistue A, Bland MJ, Mazel D. 2014. Management of multipartite genomes: the *Vibrio cholerae* model. Curr Opin Microbiol 22:120–126. doi:10.1016/j.mib.2014.10.003. 25460805

[48] Val ME, Marbouty M, de Lemos Martins F, Kennedy SP, Kemble H, Bland MJ, Possoz C, Koszul R, Skovgaard O, Mazel D. 2016. A checkpoint control orchestrates the replication of the two chromosomes of *Vibrio cholerae*. Sci Adv 2:e1501914. doi:10.1126/sciadv.1501914. 27152358 PMC4846446

[49] Soler-Bistue A, Mondotte JA, Bland MJ, Val ME, Saleh MC, Mazel D. 2015. Genomic location of the major ribosomal protein gene locus determines *Vibrio cholerae* global growth and infectivity. PLoS Genet 11:e1005156. doi:10.1371/journal.pgen.1005156. 25875621 PMC4395360

[50] Soler-Bistue A, Timmermans M, Mazel D. 2017. The proximity of ribosomal protein genes to oriC enhances *Vibrio cholerae* fitness in the absence of multifork replication. mBio 8:e00097-17. doi:10.1128/mBio.00097-17. 28246358 PMC5347342

[51] Vieira-Silva S, Rocha EP. 2010. The systemic imprint of growth and its uses in ecological (meta)genomics. PLoS Genet 6:e1000808. doi:10.1371/journal.pgen.1000808. 20090831 PMC2797632

[52] Coenye T, Vandamme P. 2005. Organisation of the S10, spc and alpha ribosomal protein gene clusters in prokaryotic genomes. FEMS Microbiol Lett 242:117–126. doi:10.1016/j.femsle.2004.10.050. 15621428

[53] Stoebe B, Kowallik KV. 1999. Gene-cluster analysis in chloroplast genomics. Trends Genet 15:344–347. doi:10.1016/s0168-9525(99)01815-6. 10461201

[54] Soler-Bistué A, Aguilar-Pierlé S, Garcia-Garcerá M, Val M-E, Sismeiro O, Varet H, Sieira R, Krin E, Skovgaard O, Comerci DJ, Rocha EPC, Mazel D. 2020. Macromolecular crowding links ribosomal protein gene dosage to growth rate in *Vibrio cholerae*. BMC Biol 18:43. doi:10.1186/s12915-020-00777-5. 32349767 PMC7191768

[55] Ochman H, Wilson AC. 1987. Evolution in bacteria: evidence for a universal substitution rate in cellular genomes. J Mol Evol 26:74–86. doi:10.1007/BF02111283. 3125340

[56] Barrick JE, Lenski RE. 2013. Genome dynamics during experimental evolution. Nat Rev Genet 14:827–839. doi:10.1038/nrg3564. 24166031 PMC4239992

[57] Kawecki TJ, Lenski RE, Ebert D, Hollis B, Olivieri I, Whitlock MC. 2012. Experimental evolution. Trends Ecol Evol 27:547–560. doi:10.1016/j.tree.2012.06.001. 22819306

[58] Lenski RE. 2017. Experimental evolution and the dynamics of adaptation and genome evolution in microbial populations. ISME J 11:2181–2194. doi:10.1038/ismej.2017.69. 28509909 PMC5607360

[59] Remigi P, Masson-Boivin C, Rocha EPC. 2019. Experimental evolution as a tool to investigate natural processes and molecular functions. Trends Microbiol 27:623–634. doi:10.1016/j.tim.2019.02.003. 30910518

[60] Lenski RE, Travisano M. 1994. Dynamics of adaptation and diversification: a 10,000-generation experiment with bacterial populations. Proc Natl Acad Sci USA 91:6808–6814. doi:10.1073/pnas.91.15.6808. 8041701 PMC44287

[61] Wibbenmeyer JA, Provenzano D, Landry CF, Klose KE, Delcour AH. 2002. *Vibrio cholerae* OmpU and OmpT porins are differentially affected by bile. Infect Immun 70:121–126. doi:10.1128/IAI.70.1.121-126.2002. 11748172 PMC127639

[62] Murphy SG, Murtha AN, Zhao Z, Alvarez L, Diebold P, Shin JH, VanNieuwenhze MS, Cava F, Dorr T. 2021. Class A penicillin-binding protein-mediated cell wall synthesis promotes structural integrity during peptidoglycan endopeptidase insufficiency in *Vibrio cholerae*. mBio 12:e03596-20. doi:10.1128/mBio.03596-20. 33824203 PMC8092314

[63] Klose KE, Mekalanos JJ. 1998. Distinct roles of an alternative sigma factor during both free-swimming and colonizing phases of the *Vibrio cholerae* pathogenic cycle. Mol Microbiol 28:501–520. doi:10.1046/j.1365-2958.1998.00809.x. 9632254

[64] Buck M, Gallegos MT, Studholme DJ, Guo Y, Gralla JD. 2000. The bacterial enhancer-dependent sigma(54) (sigma(N)) transcription factor. J Bacteriol 182:4129–4136. doi:10.1128/JB.182.15.4129-4136.2000. 10894718 PMC101881

[65] Fong JCN, Syed KA, Klose KE, Yildiz FH. 2010. Role of *Vibrio* polysaccharide (VPS) genes in VPS production, biofilm formation and *Vibrio cholerae* pathogenesis. Microbiology 156:2757–2769. doi:10.1099/mic.0.040196-0. 20466768 PMC3068689

[66] Fong JC, Yildiz FH. 2007. The rbmBCDEF gene cluster modulates development of rugose colony morphology and biofilm formation in *Vibrio cholerae*. J Bacteriol 189:2319–2330. doi:10.1128/JB.01569-06. 17220218 PMC1899372

[67] Yildiz FH, Liu XS, Heydorn A, Schoolnik GK. 2004. Molecular analysis of rugosity in a *Vibrio cholerae* O1 El Tor phase variant. Mol Microbiol 53:497–515. doi:10.1111/j.1365-2958.2004.04154.x. 15228530

[68] Yildiz FH, Schoolnik GK. 1999. *Vibrio cholerae* O1 El Tor: identification of a gene cluster required for the rugose colony type, exopolysaccharide production, chlorine resistance, and biofilm formation. Proc Natl Acad Sci USA 96:4028–4033. doi:10.1073/pnas.96.7.4028. 10097157 PMC22414

[69] Ball AS, Chaparian RR, van Kessel JC. 2017. Quorum sensing gene regulation by LuxR/HapR master regulators in *Vibrios*. J Bacteriol 199:e00105-17. doi:10.1128/JB.00105-17. 28484045 PMC5585708

[70] Ortega DR, Kjaer A, Briegel A. 2020. The chemosensory systems of *Vibrio cholerae*. Mol Microbiol 114:367–376. doi:10.1111/mmi.14520. 32347610 PMC7534058

[71] Pratt JT, McDonough E, Camilli A. 2009. PhoB regulates motility, biofilms, and cyclic di-GMP in *Vibrio cholerae*. J Bacteriol 191:6632–6642. doi:10.1128/JB.00708-09. 19734314 PMC2795287

[72] Beyhan S, Bilecen K, Salama SR, Casper-Lindley C, Yildiz FH. 2007. Regulation of rugosity and biofilm formation in *Vibrio cholerae*: comparison of VpsT and VpsR regulons and epistasis analysis of vpsT, vpsR, and hapR. J Bacteriol 189:388–402. doi:10.1128/JB.00981-06. 17071756 PMC1797413

[73] Lim B, Beyhan S, Meir J, Yildiz FH. 2006. Cyclic-diGMP signal transduction systems in *Vibrio cholerae*: modulation of rugosity and biofilm formation. Mol Microbiol 60:331–348. doi:10.1111/j.1365-2958.2006.05106.x. 16573684

[74] Beyhan S, Tischler AD, Camilli A, Yildiz FH. 2006. Transcriptome and phenotypic responses of *Vibrio cholerae* to increased cyclic di-GMP level. J Bacteriol 188:3600–3613. doi:10.1128/JB.188.10.3600-3613.2006. 16672614 PMC1482859

[75] Miller HK, Kwuan L, Schwiesow L, Bernick DL, Mettert E, Ramirez HA, Ragle JM, Chan PP, Kiley PJ, Lowe TM, Auerbuch V. 2014. IscR is essential for *Yersinia pseudotuberculosis* type III secretion and virulence. PLoS Pathog 10:e1004194. doi:10.1371/journal.ppat.1004194. 24945271 PMC4055776

[76] Dalia AB, McDonough E, Camilli A. 2014. Multiplex genome editing by natural transformation. Proc Natl Acad Sci USA 111:8937–8942. doi:10.1073/pnas.1406478111. 24889608 PMC4066482

[77] Dalia AB. 2018. Natural cotransformation and multiplex genome editing by natural transformation (MuGENT) of *Vibrio cholerae*. Methods Mol Biol 1839:53–64. doi:10.1007/978-1-4939-8685-9_6. 30047054

[78] Srivastava D, Hsieh ML, Khataokar A, Neiditch MB, Waters CM. 2013. Cyclic di-GMP inhibits *Vibrio cholerae* motility by repressing induction of transcription and inducing extracellular polysaccharide production. Mol Microbiol 90:1262–1276. doi:10.1111/mmi.12432. 24134710 PMC3881292

[79] Dymond J, Boeke J. 2012. The *Saccharomyces cerevisiae* SCRaMbLE system and genome minimization. Bioeng Bugs 3:168–171. 22572789 10.4161/bbug.19543 PMC3370935

[80] Esnault E, Valens M, Espeli O, Boccard F. 2007. Chromosome structuring limits genome plasticity in *Escherichia coli*. PLoS Genet 3:e226. doi:10.1371/journal.pgen.0030226. 18085828 PMC2134941

[81] Savic DJ, Nguyen SV, McCullor K, McShan WM. 2019. Biological impact of a large-scale genomic inversion that grossly disrupts the relative positions of the origin and terminus loci of the *Streptococcus pyogenes* chromosome. J Bacteriol 201:e00090-19. doi:10.1128/JB.00090-19. 31235514 PMC6689312

[82] Campo N, Dias MJ, Daveran-Mingot ML, Ritzenthaler P, Le Bourgeois P. 2004. Chromosomal constraints in Gram-positive bacteria revealed by artificial inversions. Mol Microbiol 51:511–522. doi:10.1046/j.1365-2958.2003.03847.x. 14756790

[83] Slager J, Kjos M, Attaiech L, Veening JW. 2014. Antibiotic-induced replication stress triggers bacterial competence by increasing gene dosage near the origin. Cell 157:395–406. doi:10.1016/j.cell.2014.01.068. 24725406

[84] Bogue MM, Mogre A, Beckett MC, Thomson NR, Dorman CJ. 2020. Network rewiring: physiological consequences of reciprocally exchanging the physical locations and growth-phase-dependent expression patterns of the *Salmonella fis* and *dps* genes. mBio 11:e02128-20. doi:10.1128/mBio.02128-20. 32900812 PMC7482072

[85] Narula J, Kuchina A, Lee DD, Fujita M, Suel GM, Igoshin OA. 2015. Chromosomal arrangement of phosphorelay genes couples sporulation and DNA replication. Cell 162:328–337. doi:10.1016/j.cell.2015.06.012. 26165942 PMC4506695

[86] Renda BA, Dasgupta A, Leon D, Barrick JE. 2015. Genome instability mediates the loss of key traits by *Acinetobacter baylyi* ADP1 during laboratory evolution. J Bacteriol 197:872–881. doi:10.1128/JB.02263-14. 25512307 PMC4325111

[87] Poltak SR, Cooper VS. 2011. Ecological succession in long-term experimentally evolved biofilms produces synergistic communities. ISME J 5:369–378. doi:10.1038/ismej.2010.136. 20811470 PMC3105725

[88] Kovacs AT, Dragos A. 2019. Evolved biofilm: review on the experimental evolution studies of *Bacillus subtilis* pellicles. J Mol Biol 431:4749–4759. doi:10.1016/j.jmb.2019.02.005. 30769118

[89] Lenski RE, Rose MR, Simpson SC. 1991. Tadler SC: long-term experimental evolution in *Escherichia coli*. I. Adaptation and divergence during 2,000 generations. Am Nat 138:1315–1341. doi:10.1086/285289.

[90] Grant NA, Abdel MA, Franklin J, Dufour Y, Lenski RE. 2021. Changes in cell size and shape during 50,000 generations of experimental evolution with *Escherichia coli*. J Bacteriol 203:e00469-20. 33649147 10.1128/JB.00469-20 PMC8088598

[91] Atolia E, Cesar S, Arjes HA, Rajendram M, Shi H, Knapp BD, Khare S, Aranda-Diaz A, Lenski RE, Huang KC. 2020. Environmental and physiological factors affecting high-throughput measurements of bacterial growth. mBio 11:e01378-20. doi:10.1128/mBio.01378-20. 33082255 PMC7587430

[92] Jun S, Si F, Pugatch R, Scott M. 2018. Fundamental principles in bacterial physiology—history, recent progress, and the future with focus on cell size control: a review. Rep Prog Phys 81:e056601. doi:10.1088/1361-6633/aaa628. PMC5897229 29313526

[93] Wiser MJ, Lenski RE. 2015. A comparison of methods to measure fitness in *Escherichia coli*. PLoS One 10:e0126210. doi:10.1371/journal.pone.0126210. 25961572 PMC4427439

[94] Concepcion-Acevedo J, Weiss HN, Chaudhry WN, Levin BR. 2015. Malthusian parameters as estimators of the fitness of microbes: a cautionary tale about the low side of high throughput. PLoS One 10:e0126915. doi:10.1371/journal.pone.0126915. 26114477 PMC4482697

[95] Stutzmann S, Blokesch M. 2020. Comparison of chitin-induced natural transformation in pandemic *Vibrio cholerae* O1 El Tor strains. Environ Microbiol 22:4149–4166. doi:10.1111/1462-2920.15214. 32860313 PMC7693049

[96] Kamareddine L, Wong ACN, Vanhove AS, Hang S, Purdy AE, Kierek-Pearson K, Asara JM, Ali A, Morris JG, Jr, Watnick PI. 2018. Activation of *Vibrio cholerae* quorum sensing promotes survival of an arthropod host. Nat Microbiol 3:243–252. doi:10.1038/s41564-017-0065-7. 29180725 PMC6260827

[97] Blow NS, Salomon RN, Garrity K, Reveillaud I, Kopin A, Jackson FR, Watnick PI. 2005. *Vibrio cholerae* infection of *Drosophila melanogaster* mimics the human disease cholera. PLoS Pathog 1:e8. doi:10.1371/journal.ppat.0010008. 16201020 PMC1238743

[98] Raeside C, Gaffe J, Deatherage DE, Tenaillon O, Briska AM, Ptashkin RN, Cruveiller S, Medigue C, Lenski RE, Barrick JE, Schneider D. 2014. Large chromosomal rearrangements during a long-term evolution experiment with *Escherichia coli*. mBio 5:e01377-14. doi:10.1128/mBio.01377-14. 25205090 PMC4173774

[99] Lang KS, Merrikh H. 2018. The clash of macromolecular titans: replication-transcription conflicts in bacteria. Annu Rev Microbiol 72:71–88. doi:10.1146/annurev-micro-090817-062514. 29856930 PMC6233710

[100] Wang Z, Hang S, Purdy AE, Watnick PI. 2013. Mutations in the IMD pathway and mustard counter *Vibrio cholerae* suppression of intestinal stem cell division in *Drosophila*. mBio 4:e00337-13. doi:10.1128/mBio.00337-13. 23781070 PMC3684835

[101] Colin R, Ni B, Laganenka L, Sourjik V. 2021. Multiple functions of flagellar motility and chemotaxis in bacterial physiology. FEMS Microbiol Rev 45:fuab038. doi:10.1093/femsre/fuab038. 34227665 PMC8632791

[102] Ni B, Ghosh B, Paldy FS, Colin R, Heimerl T, Sourjik V. 2017. Evolutionary remodeling of bacterial motility checkpoint control. Cell Rep 18:866–877. doi:10.1016/j.celrep.2016.12.088. 28122238 PMC5289928

[103] Ni B, Colin R, Link H, Endres RG, Sourjik V. 2020. Growth-rate dependent resource investment in bacterial motile behavior quantitatively follows potential benefit of chemotaxis. Proc Natl Acad Sci USA 117:595–601. doi:10.1073/pnas.1910849117. 31871173 PMC6955288

[104] Izard J, Gomez BC, Ropers D, Lacour S, Song X, Yang Y, Lindner AB, Geiselmann J, de Jong H. 2015. A synthetic growth switch based on controlled expression of RNA polymerase. Mol Syst Biol 11:840. doi:10.15252/msb.20156382. 26596932 PMC4670729

[105] Gyorfy Z, Draskovits G, Vernyik V, Blattner FF, Gaal T, Posfai G. 2015. Engineered ribosomal RNA operon copy-number variants of *E. coli* reveal the evolutionary trade-offs shaping rRNA operon number. Nucleic Acids Res 43:1783–1794. doi:10.1093/nar/gkv040. 25618851 PMC4330394

[106] Heidelberg JF, Eisen JA, Nelson WC, Clayton RA, Gwinn ML, Dodson RJ, Haft DH, Hickey EK, Peterson JD, Umayam L, Gill SR, Nelson KE, Read TD, Tettelin H, Richardson D, Ermolaeva MD, Vamathevan J, Bass S, Qin H, Dragoi I, Sellers P, McDonald L, Utterback T, Fleishmann RD, Nierman WC, White O, Salzberg SL, Smith HO, Colwell RR, Mekalanos JJ, Venter JC, Fraser CM. 2000. DNA sequence of both chromosomes of the cholera pathogen *Vibrio cholerae*. Nature 406:477–483. doi:10.1038/35020000. 10952301 PMC8288016

[107] Martinez-Gil M, Goh KGK, Rackaityte E, Sakamoto C, Audrain B, Moriel DG, Totsika M, Ghigo J-M, Schembri MA, Beloin C. 2017. YeeJ is an inverse autotransporter from *Escherichia coli* that binds to peptidoglycan and promotes biofilm formation. Sci Rep 7:11326. doi:10.1038/s41598-017-10902-0. 28900103 PMC5595812

[108] Li H, Durbin R. 2009. Fast and accurate short read alignment with Burrows-Wheeler transform. Bioinformatics 25:1754–1760. doi:10.1093/bioinformatics/btp324. 19451168 PMC2705234

[109] Li H, Handsaker B, Wysoker A, Fennell T, Ruan J, Homer N, Marth G, Abecasis G, Durbin R. 1000 Genome Project Data Processing Subgroup. 2009. The sequence alignment/map format and SAMtools. Bioinformatics 25:2078–2079. doi:10.1093/bioinformatics/btp352. 19505943 PMC2723002

[110] DePristo MA, Banks E, Poplin R, Garimella KV, Maguire JR, Hartl C, Philippakis AA, del Angel G, Rivas MA, Hanna M, McKenna A, Fennell TJ, Kernytsky AM, Sivachenko AY, Cibulskis K, Gabriel SB, Altshuler D, Daly MJ. 2011. A framework for variation discovery and genotyping using next-generation DNA sequencing data. Nat Genet 43:491–498. doi:10.1038/ng.806. 21478889 PMC3083463

[111] Koboldt DC, Zhang Q, Larson DE, Shen D, McLellan MD, Lin L, Miller CA, Mardis ER, Ding L, Wilson RK. 2012. VarScan 2: somatic mutation and copy number alteration discovery in cancer by exome sequencing. Genome Res 22:568–576. doi:10.1101/gr.129684.111. 22300766 PMC3290792

[112] Cingolani P, Platts A, Wang Le L, Coon M, Nguyen T, Wang L, Land SJ, Lu X, Ruden DM. 2012. A program for annotating and predicting the effects of single nucleotide polymorphisms, SnpEff: SNPs in the genome of *Drosophila melanogaster* strain w1118; iso-2; iso-3. Fly 6:80–92. doi:10.4161/fly.19695. 22728672 PMC3679285

[113] Nurk S, Bankevich A, Antipov D, Gurevich AA, Korobeynikov A, Lapidus A, Prjibelski AD, Pyshkin A, Sirotkin A, Sirotkin Y, Stepanauskas R, Clingenpeel SR, Woyke T, McLean JS, Lasken R, Tesler G, Alekseyev MA, Pevzner PA. 2013. Assembling single-cell genomes and mini-metagenomes from chimeric MDA products. J Comput Biol 20:714–737. doi:10.1089/cmb.2013.0084. 24093227 PMC3791033

[114] Galardini M, Biondi EG, Bazzicalupo M, Mengoni A. 2011. CONTIGuator: a bacterial genomes finishing tool for structural insights on draft genomes. Source Code Biol Med 6:11. doi:10.1186/1751-0473-6-11. 21693004 PMC3133546

[115] Carver TJ, Rutherford KM, Berriman M, Rajandream MA, Barrell BG, Parkhill J. 2005. ACT: the Artemis comparison tool. Bioinformatics 21:3422–3423. doi:10.1093/bioinformatics/bti553. 15976072

[116] de Lemos Martins F, Fournes F, Mazzuoli MV, Mazel D, Val ME. 2018. *Vibrio cholerae* chromosome 2 copy number is controlled by the methylation-independent binding of its monomeric initiator to the chromosome 1 crtS site. Nucleic Acids Res 46:10145–10156. 30184118 10.1093/nar/gky790 PMC6212839

[117] Schneider CA, Rasband WS, Eliceiri KW. 2012. NIH Image to ImageJ: 25 years of image analysis. Nat Methods 9:671–675. doi:10.1038/nmeth.2089. 22930834 PMC5554542

[118] Hall BG, Acar H, Nandipati A, Barlow M. 2014. Growth rates made easy. Mol Biol Evol 31:232–238. doi:10.1093/molbev/mst187. 24170494

