# Supplementary material for: Chromosomal Position of Ribosomal Protein Genes Affects Long-Term Evolution of Vibrio cholerae
Source: mBio. 2023 Mar 2;14(2):e03432-22. doi: 10.1128/mbio.03432-22 (PMC10127744; doi:10.1128/mbio.03432-22)
Supplement: TABLE S1 [file mbio.03432-22-s0006.pdf]

Name	Relevant genotype and/or phenotype	Reference
Escherichia coli		
XL-Blue	*recA1 endA1 gyrA96 thi-1 hsdR17 supE44 relA1 lac* [F´ *proAB lacI^q^ ZΔM15* Tn10 (Tet^R)]	
Vibrio cholerae		
Wild type	N16961::mTn*7hapR*$^+$ Δ*lacZ*. Er^S, Gn^R and Cm^S.	Val et al. 2012
Parental	PGB-A192::*attB'-lox66-dfrB1-lox71* inserted in the intergenic region between VC1508-VC1509. Er^S, Gn^R and Cm^R.	Soler-Bistué et al. 2015
S10Tnp-35	S10 relocated next to its original location in the intergenic region between VC2536-VC2537.	Soler-Bistué et al. 2015
S10Tnp-1120	S10 relocated near the *dif* region of chromosome 1 in the intergenic region VC1508-VC1509. Er^S, Gn^R and Cm^R.	Soler-Bistué et al. 2015
S10TnpC2+479	S10 relocated near the *dif* sequence of chromosome 2 in the intergenic region between VCA0543-VCA0544.	Soler-Bistué et al. 2015
FG-G250-2	Fast-growing clone selected from population 2 after 250 generations of evolution.	This work
FG-G250-3	Fast-growing clone selected from population 3 after 250 generations of evolution.	This work
FG-G250-7	Fast-growing clone selected from population 7 after 250 generations of evolution.	This work
FG-G250-10	Fast-growing clone selected from population 10 after 250 generations of evolution.	This work
FG-G250-11	Fast-growing clone selected from population 11 after 250 generations of evolution.	This work
G1000-P1-S	Smooth-colony clone isolated from population 1 after 1000 generations of evolution.	This work
G1000-P1-R	Rugose-colony clone isolated from population 1 after 1000 generations of evolution.	This work
G1000-P10-S	Smooth-colony clone isolated from population 10 after 1000 generations of evolution.	This work
G1000-P10-R	Rugose-colony clone isolated from population 10 after 1000 generations of evolution.	This work

Parental MuGENT	N16961::mTn*7hapR*$^+$ Δ*lacZ*. Er^S, Gn^R and Cm^S. The $Spec^R$ cassette was inserted in a neutral region between VC1902-VC1903.	This work
*flrA**	Parental MuGENT *flrA*^{FS} $\Delta(C)_5$ at base number 2,163,111. $Spec^R$ cassette inserted in neutral region between VC1902-VC1903.	This work
*flrB**	Parental MuGENT *flrB** ΔGCGAGTG at base number 2,162,019 . $Spec^R$ cassette inserted in neutral region between VC1902-VC1903.	This work
*mgtE**	Parental MuGENT *MgtE** point mutation, G for C at chromosome II position 765560. The $Spec^R$ cassette was inserted in a neutral region between VC1902-VC1903.	This work
