# Supplementary figures and images for: Chromosomal Position of Ribosomal Protein Genes Affects Long-Term Evolution of Vibrio cholerae
Source: mBio. 2023 Mar 2;14(2):e03432-22. doi: 10.1128/mbio.03432-22 (PMC10127744; doi:10.1128/mbio.03432-22)

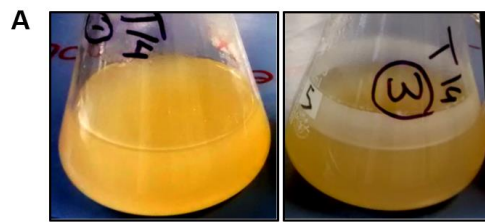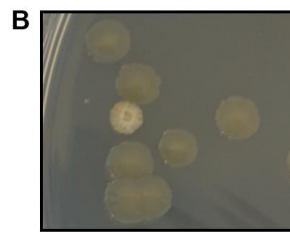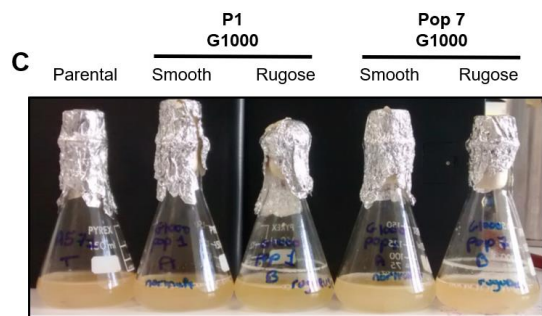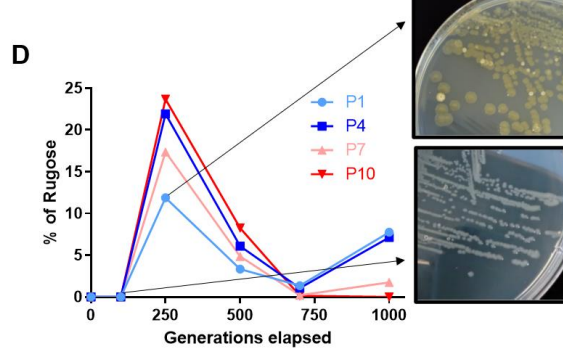

Supplement: FIG S1 [file mbio.03432-22-s0001.pdf]

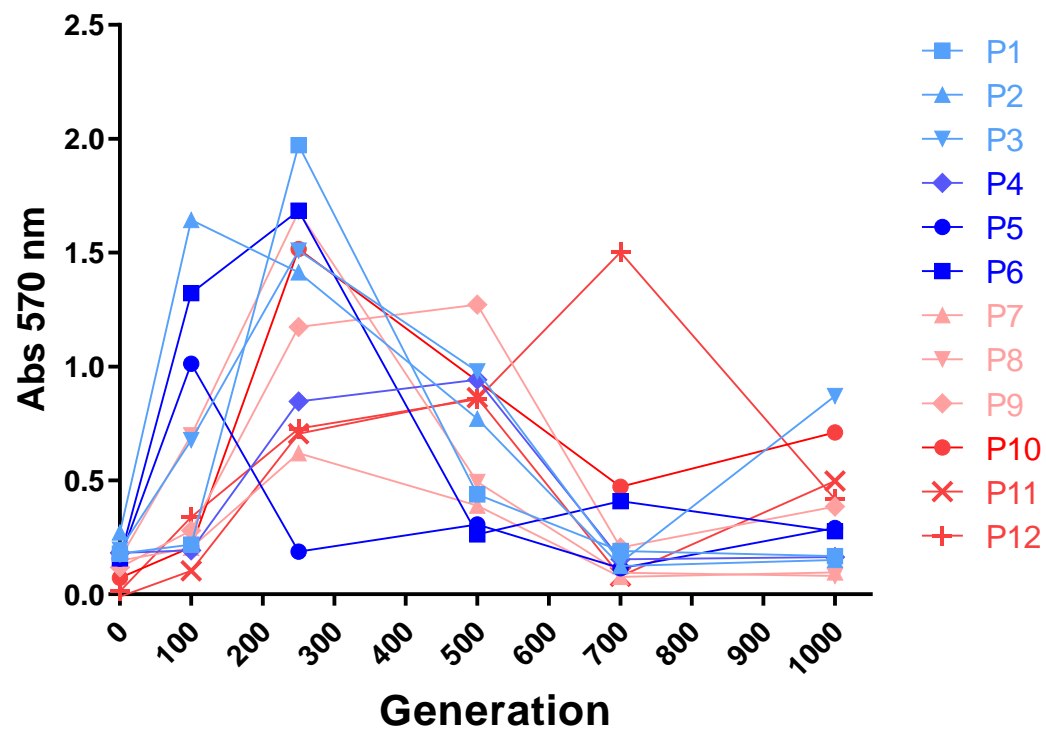

Supplement: FIG S2 [file mbio.03432-22-s0002.pdf]

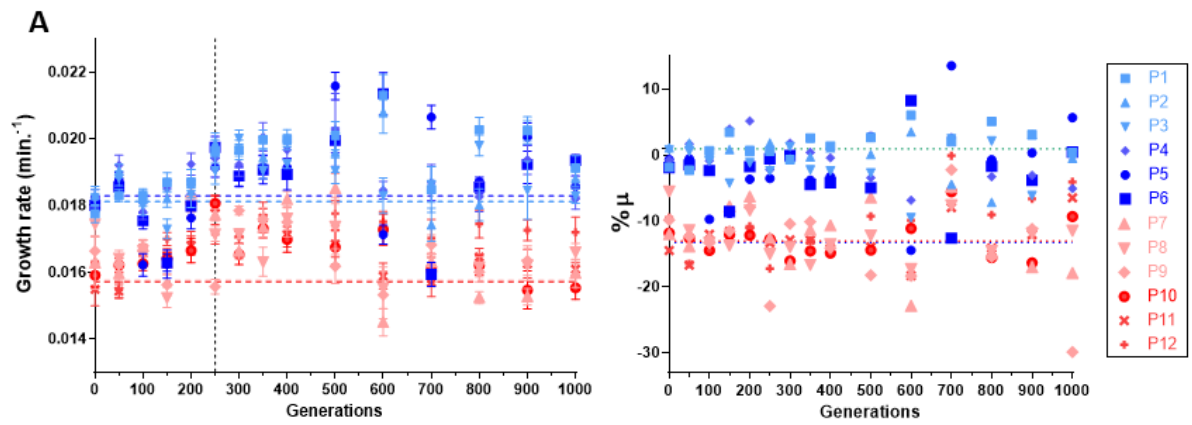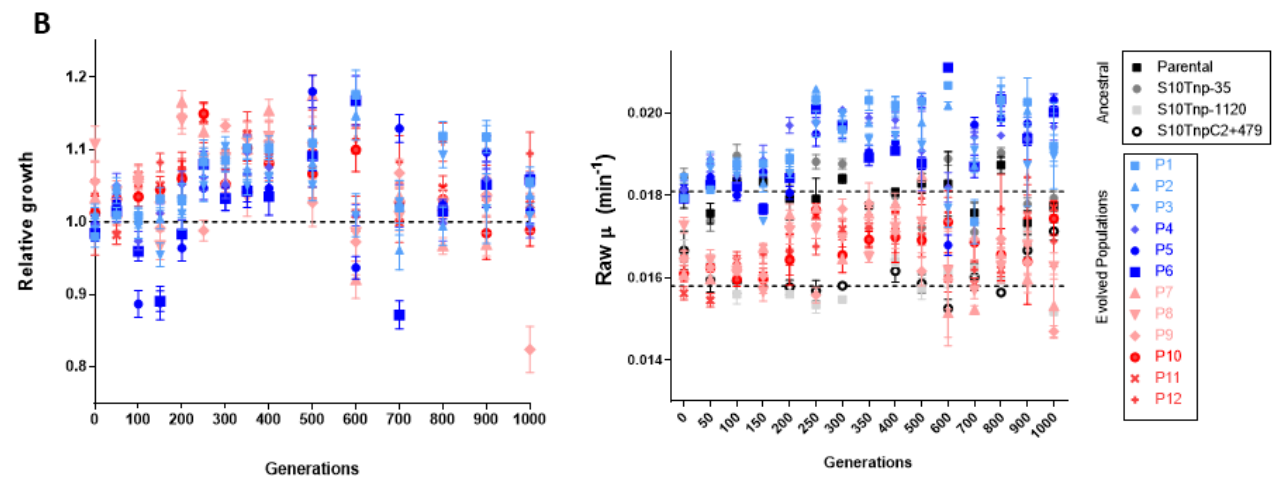

Supplement: FIG S3 [file mbio.03432-22-s0003.pdf]

A

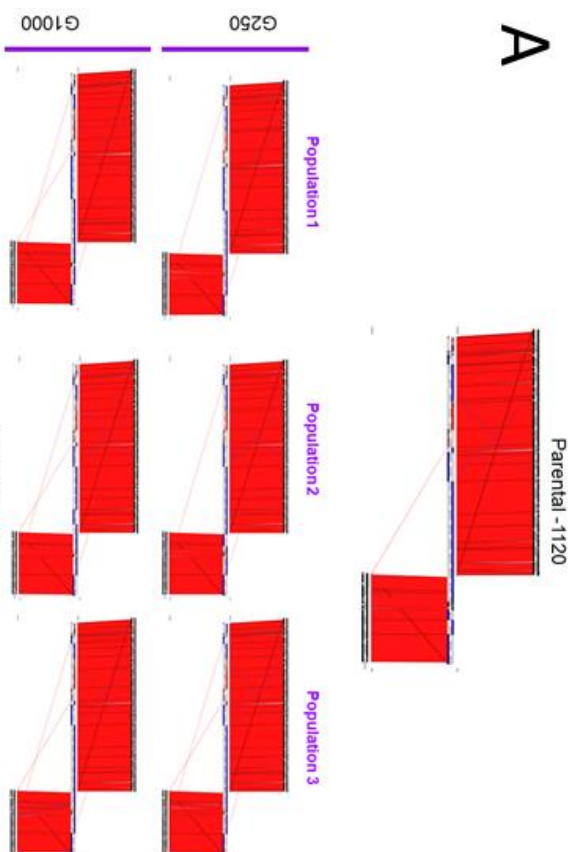

C

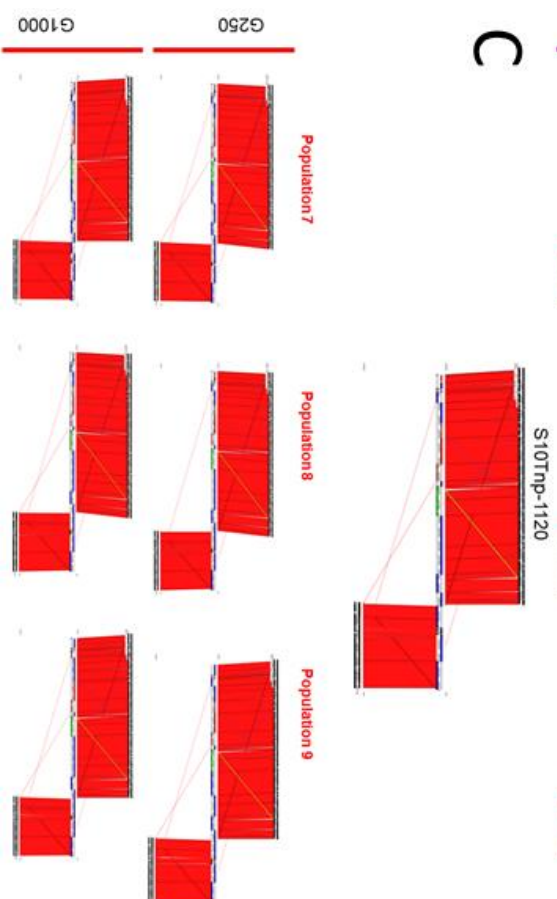

B

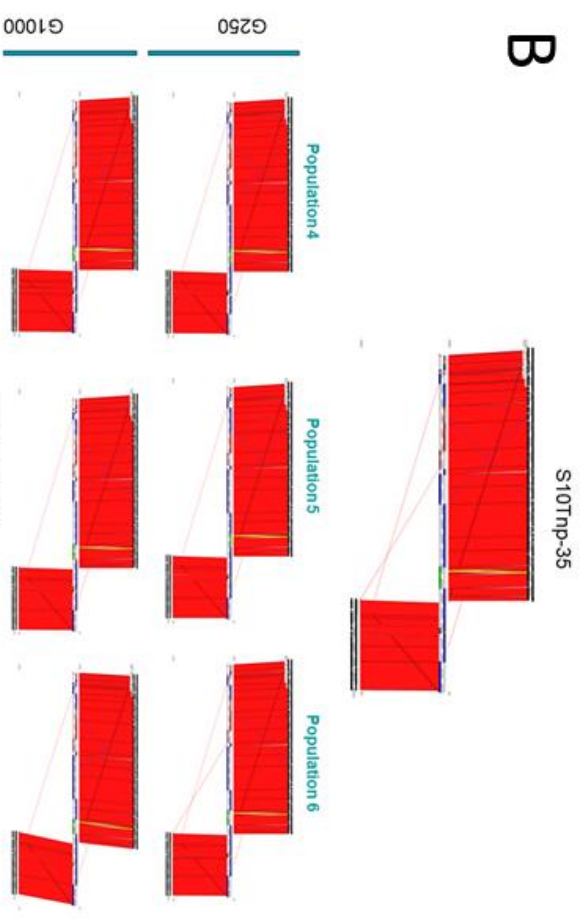

D

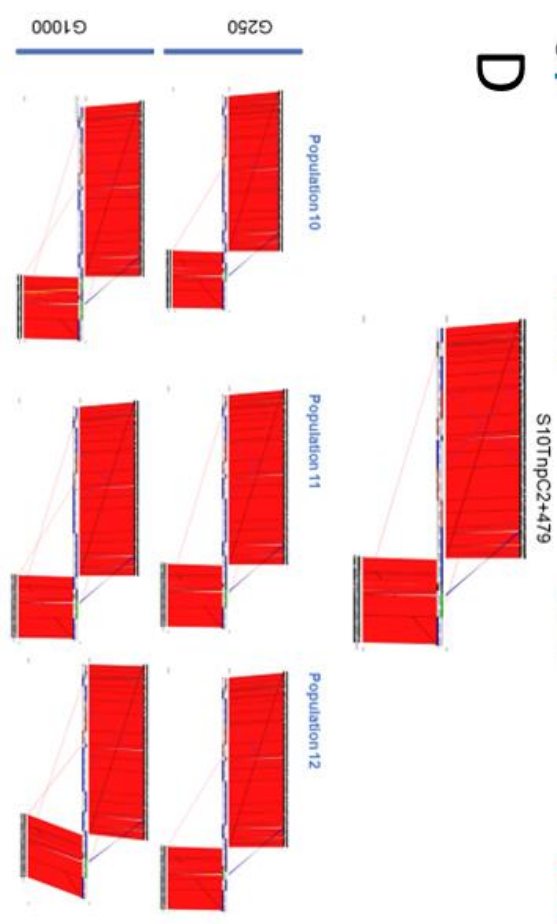

Supplement: FIG S4 [file mbio.03432-22-s0004.pdf]

FG-G250-2

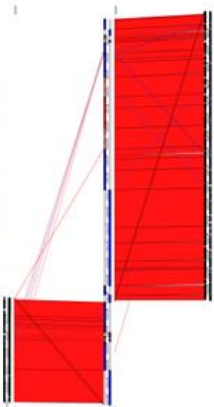

G250-FG-3

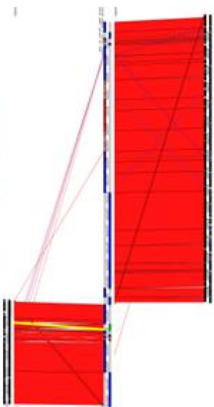

G250-FG-7

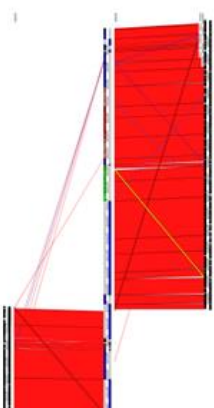

FG-G250-10

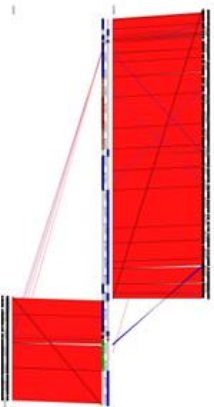

G250-FG-11

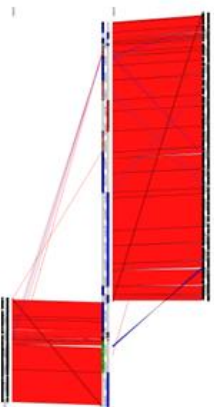

G1000-1-S

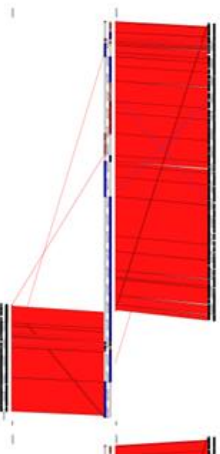

G1000-1-R

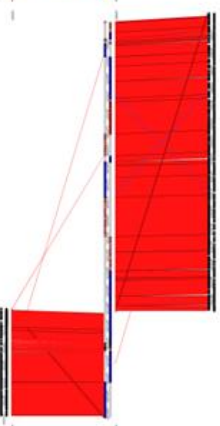

G1000-10-S

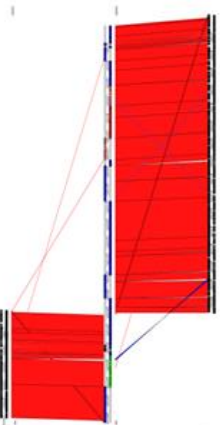

G1000-10-R

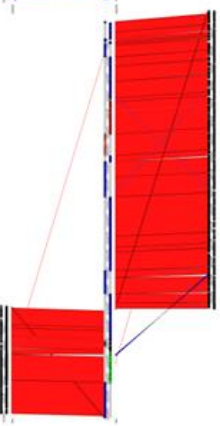

Supplement: FIG S5 [file mbio.03432-22-s0005.pdf]
