# Supplementary material for: Chromosomal Position of Ribosomal Protein Genes Affects Long-Term Evolution of Vibrio cholerae
Source: mBio. 2023 Mar 2;14(2):e03432-22. doi: 10.1128/mbio.03432-22 (PMC10127744; doi:10.1128/mbio.03432-22)
Supplement: TABLE S2 [file mbio.03432-22-s0007.pdf]

| Primer name | Sequence (5’-3’) | Target |
|---|---|---|
| rctB_qPCR_F | GAAGTCTCTGAAGCCGCGATTG | *rctB* |
| rctB_qPCR5-R | CCTCTTCGGAAGCGGTGATG | *rctB* |
| Tgt4-1 | ATCGGTTGCGTCACCAAATG | Superintegron (VCA0543) |
| Tgt4-4 | GCATTCGATTCGTCGTTTGG | Superintegron (VCA0544) |
| MUGENTT_FlrAFS_Seq1 | TTACGAGGAGAGGCATTGTG | VC2137//*flrA* |
| MUGENTT_ FlrAFS_1 | TCGGTCTTCGCCACTTTATC | VC2137//*flrA* |
| MUGENTT_ FlrAFS_2 | CAAGCGTTAGAAGCGCAATAGTGGCGAGAGCGGCAGAC | VC2137//*flrA* |
| MUGENTT_ FlrAFS _3 | TGCCGCTCTCGCCACTATTGCGCTTCTAACGCTTGGT | VC2137//*flrA* |
| MUGENTT_ FlrAFS _4 | TTGACTGCCGCTGAAGTGGG | VC2137//*flrA* |
| MUGENTT_ FlrA FS_Seq2 | CGCAAATGCTCGATGATGTG | VC2137//*flrA* |
| F_Verif_FlrAdelC | TCTCGCCACTATTGCGCTTC | VC2137//*flrA* |
| R_Verif_ FlrAdelC | CCATATCCCGTTGCTGGTTG | VC2137/*flrA* |
| MUGENTT_FlrB_Seq1 | TATCCGGCTCACGTACCCAG | VC2136/*flrB* |
| MUGENTT_FlrB_1 | CATCGGCCACAGTCTATTACC | VC2136/*flrB* |
| MUGENTT_FlrB_2 | CAAAATCATGGAACCCTTTAAGGTACAGGCCTTGGCTTA | VC2136/*flrB* |
| MUGENTT_FlrB_3 | TAAGCCAAGGCCTGTACCTTAAAGGGTTCCATGATTTTGT | VC2136/*flrB* |
| MUGENTT_FlrB_4 | CCTTGTTGGGCAAAGCATGG | VC2136/*flrB* |
| MUGENTT_FlrB_Seq2 | CCATATCCCGTTGCTGGTTG | VC2136/*flrB* |
| R_Verif_FlrB | ATCTCGCTGCGCAATGGACG | VC2136/*flrB* |
| F_WT_FlrB | AGGCCTGTACCTTGTGAGCGAGTG | VC2136/*flrB* |
| MUGENTT_MgtE_Seq1 | CACACCTGACACCCTAGATG | VCA0818/*mgtE* |
| MUGENT_MgtE_1 | TTTCTGTACGCCCTTGATGC | VCA0818/*mgtE* |
| MUGENT_MgtE_2 | AAGCCGACCACATCGGTCACGGGTGGTGAG | VCA0818/*mgtE* |
| MUGENT_MgtE_3 | CAGGTTCGGTTATTCTCACCACCCGTGACC | VCA0818/*mgtE* |

| | | |
|---|---|---|
| MUGENT_MgtE_4 | GCAGCGTTGTAGCCGTAATG | VCA0818/*mgtE* |
| MUGENT_MgtE_Seq2 | GCCGCAGACTCTTTGTCTAC | VCA0818/*mgtE* |
| F_Verif_MgtE | TTCGGTTATTCTCACCACTC | VCA0818/*mgtE* |
| R_Verif_MgtE | ACTCGGAGAGGATCAGATAC | VCA0818/*mgtE* |
