# Supplementary material for: Chromosomal Position of Ribosomal Protein Genes Affects Long-Term Evolution of Vibrio cholerae
Source: mBio. 2023 Mar 2;14(2):e03432-22. doi: 10.1128/mbio.03432-22 (PMC10127744; doi:10.1128/mbio.03432-22)
Supplement: TABLE S3 [file mbio.03432-22-s0008.pdf]

Sequenced Sample	Total Reads	Sequencing Depth	Read Lenght	Reads Mapped on Chr1	Reads Mapped on Chr1	Mapping depth Chr 1 (X)	Mapping depth Chr 2 (X)
Parental -1120	17.626.690	437	100	13.321.904	4.122.883	450	384
S10Tnp-35	14.918.414	370	100	11.825.096	2.876.546	399	268
S10Tnp-1120	5.218.668	129	100	3.919.111	918.582	132	86
S10TnpC2+479	4.397.288	327	300	3.196.177	1.154.727	324	323
G250_P1	36.963.402	1146	125	26.806.911	9.914.986	1132	1156
G250_P2	22.550.552	699	125	16.335.823	6.039.280	690	704
G250_P3	1.842.358	69	151	1.319.642	484.313	67	68
G250_P4	26.289.516	815	125	18.972.490	7.062.682	801	823
G250_P5	31.369.490	972	125	22.768.106	8.374.077	961	976
G250_P6	26.670.034	827	125	19.227.845	7.115.354	812	829
G250_P7	17.907.510	555	125	12.918.038	4.813.371	545	561
G250_P8	23.993.272	744	125	17.282.528	6.490.831	730	757
G250_P9	23.742.656	736	125	17.375.636	6.176.645	733	720
G250_P10	1.604.940	60	151	1.148.311	423.280	59	60
G250_P11	29.833.280	925	125	21.359.819	7.938.893	902	925
G250_P12	23.512.082	729	125	16.976.728	6.237.071	717	727
G1000_P1	18.622.052	577	125	13.638.724	4.735.656	576	552
G1000_P2	20.077.018	622	125	14.737.088	5.140.841	622	599
G1000_P3	2.102.078	79	151	1.515.747	538.571	77	76
G1000_P4	23.729.064	735	125	17.298.765	6.135.493	730	715
G1000_P5	22.583.454	700	125	16.384.764	5.851.284	692	682
G1000_P6	24.523.816	760	125	17.797.257	6.334.684	751	738
G1000_P7	22.211.820	688	125	16.191.623	5.787.108	684	675
G1000_P8	18.652.162	578	125	13.565.722	4.840.662	573	564
G1000_P9	29.662.968	919	125	21.630.567	7.789.654	913	908
G1000_P10	1.666.826	62	151	1.192.975	425.774	61	60
G1000_P11	21.553.942	668	125	15.626.231	5.689.946	660	663
G1000_P12	37.454.614	1161	125	27.165.998	9.789.920	1147	1141
FG-G250-2	14.383.706	385	108	11.544.204	2.716.131	421	274
FG-G250-3	16.976.140	455	108	13.583.826	3.239.258	495	326
FG-G250-7	11.775.216	315	108	9.222.812	2.372.905	336	239
FG-G250-10	13.228.832	354	108	10.518.652	2.506.843	384	252
FG-G250-11	13.716.730	367	108	10.938.111	2.591.894	399	261
G1000_P1_A	14.792.978	458	125	10.829.949	3.876.524	457	452
G1000_P1_B	25.030.204	776	125	18.166.283	6.400.725	767	746
G1000_P12_A	16.669.538	517	125	12.226.844	4.361.155	516	508
G1000_P12_B	19.446.346	603	125	14.165.001	5.039.105	598	587
